# Overconfidence in ability to discern cancer misinformation: a conceptual replication and extension

**DOI:** 10.1093/hcr/hqaf017

**Published:** 2025-07-14

**Authors:** Benjamin Lyons, Andy J King, Kimberly A Kaphingst

**Affiliations:** Department of Communication, University of Utah, Salt Lake City, United States; Cancer Control and Population Sciences, Huntsman Cancer Institute, Salt Lake City, United States; Department of Communication, University of Utah, Salt Lake City, United States; Cancer Control and Population Sciences, Huntsman Cancer Institute, Salt Lake City, United States; Department of Communication, University of Utah, Salt Lake City, United States; Cancer Control and Population Sciences, Huntsman Cancer Institute, Salt Lake City, United States

**Keywords:** overconfidence, misinformation, health communication, digital trace data, behavioral data

## Abstract

This study conducts a conceptual replication and extension of prior research on overconfidence in discerning political misinformation, shifting focus to health. Using data from a national survey of American adults linked to web-browsing records (*N*=593), we investigated the prevalence and correlates of overconfidence in this domain. Consistent with earlier findings, overconfidence was most pronounced among low-performing individuals, replicating the “Dunning–Kruger Effect.” However, decomposition analyses revealed that the associations between overconfidence and behavioral correlates—exposure to low-credibility health websites and belief in cancer-related misinformation—are primarily accounted for by deficits in actual discernment ability rather than a lack of metacognitive insight. This replication highlights the robustness of the link between poor discernment and behavioral outcomes across domains. These results highlight the need for interventions that improve evaluation skills or address underlying dispositional factors, such as distrust in science and conspiracist worldviews.

## Public significance statement

Many Americans overestimate their ability to identify cancer misinformation online. However, this study shows that poor skill itself—not poor insight into one’s skill level—best explains risky behaviors like visiting unreliable websites. Improving people’s evaluation skills may help reduce the spread and influence of health misinformation.

Many individuals struggle to identify unreliable information and may overestimate their ability to do so (Dunning, 2011; Lyons et al., 2021).^1^ This lack of metacognitive insight can lead people to unwittingly engage with more low-credibility content (Lyons et al., 2021). While this has been documented in political contexts, less is known about how overconfidence operates in health domains such as cancer misinformation, which remains widespread (Johnson et al., 2022). This issue matters: belief in alternative cancer treatments has been tied to increased mortality (Johnson et al., 2018), and misinformation about screening procedures may reduce uptake (Swire-Thompson & Johnson, 2024).

How pervasive is overconfidence in *health* contexts? Are there potential behavioral consequences? We address these questions using data from a national survey of Americans linked to web-browsing data (*N*=593), replicating and extending the design of Lyons et al. (2021). That study, based on national surveys conducted during the 2018 U.S. midterms, measured participants’ political news discernment, confidence in those abilities, belief in political falsehoods, and passively tracked exposure to dubious political news sites. We adapt this approach to the health domain, focusing on respondents’ perceived and actual ability to distinguish true from false cancer-related information. This allows us to assess the prevalence of overconfidence in this topic and its relationship to belief in cancer misperceptions and exposure to low-credibility health sources.

Although most respondents are at least somewhat overconfident on a relative scale (compared to peers), respondents show little to no overconfidence on an absolute scale (estimating the number of items answered correctly). However, a “Dunning–Kruger” pattern (Dunning, 2011)—with significant overconfidence among the worst performers—is still evident. While we find that relative overconfidence is associated with exposure to low-credibility health sites and belief in cancer misperceptions, decomposition of overconfidence’s constituent terms suggests that it is a lack of actual ability, more so than a lack of awareness about said ability deficits, that accounts for this. All told, our findings and those of the replicated study jointly underline the significance of discernment skills in explaining exposure to and belief in topical misperceptions across domains of politics and health, and hold implications for the design of interventions in these spaces. The rationale for a conceptual replication and our preregistered research questions are outlined below.^2^

### The value of conceptual replication

Conceptual replications play a critical role in evaluating the general validity of theoretical predictions (Stroebe & Strack, 2014), allowing researchers to test whether an effect is robust to variations in study design. This study replicates important findings on overconfidence and its behavioral correlates, extending prior work to the context of cancer-related misinformation—a domain with significant public health implications. There are key differences between politics and health that underlie the importance of this replication. First, task familiarity and fluency may differ, which could modulate metacognitive calibration. People may feel more “fluent” reading political headlines framed in ways that match preexisting schemas (Sparks & Hmielowski, 2023), while health headlines may feel more novel or technical, lowering fluency and increasing uncertainty (Daraz et al., 2018). This could reduce overconfidence (especially on absolute measures). Politics also produces strong identity-based motivations (Mason, 2018), which could inflate overconfidence because perceived skill protects one’s identity. For health, motivations are often driven by safety concerns or distrust, rather than identity. There may be less incentive to maintain belief in superiority, so metacognitive distortions may be weaker. Similarly, these domains vary in self-relevance. In politics, beliefs are often abstract and symbolic, so confidence can be performative. Health beliefs have personal consequences which can foster greater caution. In sum, overconfidence in political domains may be instrumental (seeing one’s self as savvy) while in health it may be more incidental (a byproduct of performance, not active self-enhancement).

Thus, there are both theoretical and practical motivations for this replication. Theoretically, cancer content may represent a legitimate boundary condition for Dunning–Kruger effects due to its different motivational structure and higher epistemic uncertainty. Practically, health misinformation is a high-stakes domain, and understanding whether overconfidence contributes to harmful behavior can help inform the design and targeting of interventions and public communication strategies.

### Exploring the Dunning–Kruger Effect in the context of cancer information

Expanding on previous research on perceptual biases in self-assessment, and political news discernment in particular (Lyons et al., 2021), this study examines the presence of the Dunning–Kruger Effect (DKE) in the context of cancer information discernment. To our knowledge, this is a novel application of this theoretical framework to the cancer context. The DKE highlights a prevalent pattern where individuals with lower competence in various intellectual pursuits fail to recognize their own deficiencies, while highly competent individuals tend to slightly underestimate their abilities relative to others. This phenomenon appears consistently across various evaluation methods, including comparative self-assessments (ratings relative to peers) and absolute self-assessments (Dunning, 2011), both of which are used in the present study.

The DKE posits a dual challenge for low performers: their lack of expertise not only leads to mistakes but also inhibits their ability to identify these errors or accurately assess others’ capabilities. Research has shown that individuals in the lowest quartile of performance often exhibit the most inflated self-perceptions. These distortions are not simply efforts to preserve self-esteem but reflect genuine overconfidence in their abilities (Dunning, 2011). Building on this body of work, this study seeks to address the following research question:

**Research Question 1**. To what extent do people who are least accurate at distinguishing between accurate and inaccurate cancer information overrate their ability?

As others have noted, the DKE may have significant consequences for individual behavior (Lyons et al., 2021). Within the context of cancer information discernment, overconfidence may correlate with different behavioral tendencies. Firstly, we might anticipate a positive relationship between overconfidence and visits to low-credibility health websites. The DKE suggests that individuals with limited discernment abilities are less capable of identifying questionable information when exposed to it and are often unaware of this limitation, increasing the likelihood of unintentional exposure to unreliable content. Alternatively, overconfidence can manifest as a sense of invulnerability, where perceived expertise reduces the motivation to engage in precautionary measures—such as critically evaluating the credibility of websites. Indeed, in recent work, overconfidence in (political) news discernment has been found to correlate with visits to dubious political news (Lyons et al., 2021). Still, as we expand to the domain of cancer and health information more broadly, our research is exploratory. As such, we propose the following research question:

**Research Question 2**. Is overconfidence related to low-credibility health site exposure?

Moreover, overconfidence might reduce scrutiny of questionable information, as high levels of confidence often correlate with reduced reflective thinking (Pennycook et al., 2017; Thompson et al., 2011). Consequently, overconfident individuals may exhibit a greater tendency to accept false claims as true (Salovich & Rapp, 2020).^3^ Our final research question asks:

**Research Question 3.** Is overconfidence related to misperceptions about cancer?

## Methods

Survey data (total *N*=1,200; see below for rationale for *N*=593 for this replication report) were collected by YouGov from October 25 to November 21, 2023. Respondents were selected by YouGov’s matching and weighting algorithm to approximate the demographic attributes of the U.S. population (see online supplementary material, Appendix A for a full description of sampling procedures). Our participants closely resemble the U.S. population in demographics (see online supplementary material, Table S1).

### Measures

#### Headline accuracy discernment

Our primary measures are based on headline evaluations.^4^ A randomized half of the total sample provided accuracy ratings, and the other half provided sharing intentions in response to accurate and inaccurate cancer information. We focus on accuracy ratings in this paper and, thus, limit analysis to those respondents (*N*=593 respondents). Respondents were exposed to and evaluated a total of 18 headlines relating to cancer published in online outlets: six inaccurate headlines (randomly drawn from a pool of 16) and 12 accurate headlines (randomly drawn from a pool of 44). Detail on headlines is presented in online supplementary material, Appendix A.3. Perceived accuracy for each item is measured on a four-point scale (“Not at all accurate” (1) to “Very accurate” (4)). We calculate a measure of performance in distinguishing accurate from inaccurate—*discernment—*as mean (accurate item accuracy)−mean (inaccurate item accuracy).

#### Confidence measures

After the headline evaluation task, we collected two measures of confidence. First, we measured relative confidence (self-rated percentile): “How do you think you compare to other Americans in how well you performed in this study at recognizing news that is inaccurate? Please respond using the scale below, where 1 means you’re at the very bottom (worse than 99% of people) and 100 means you’re at the very top (better than 99% of people),” (1–100). “Don’t know” was also a response option, which we treat as missing (*n*=130 DKs) so we use 463 responses to this item below. Next, we assessed absolute confidence (confidence [number correct]): “You just made 18 news evaluations. How many do you think you got right?” (0–18).

#### Overconfidence measures

We then computed two overconfidence measures, extending Lyons et al. (2021) as there is potential for diverging findings between the measures. Some have argued that the DKE pattern is found regardless of using relative or absolute measures of confidence (that is, eliciting either self-placement relative to others or absolute performance metrics; Ehrlinger et al., 2008). Others show that on easy tasks, people tend to underestimate their actual performances but believe they are better than others (Moore & Healy, 2008). Others argue that relative overconfidence is predicted by cognitive abilities or dispositions, while an absolute measure is not (despite their intercorrelation), suggesting the latter could be less a systematic bias and more reflective of noise (Duttle, 2016). Hence, it is important to examine multiple forms of overconfidence: overplacement (our measure of relative overconfidence), which is more typically used in DKE studies, and overestimation (our measure of absolute overconfidence).

*Relative overconfidence* (i.e., overplacement) is computed as (self-rated percentile)−(actual percentile in discernment). Actual percentile in discernment is calculated by taking discernment scores, ordering respondents and calculating their percentile. Each respondent is scored on a scale ranging from 1 to 100 based on their performance, where a score of 1 means that 99% of respondents performed better and a score of 99 means that they performed better than 99% of respondents.

*Absolute overconfidence* (i.e., overestimation) is calculated as (confidence [number correct])−(actual number correct). Actual number correct collapses responses on the 4-point accuracy scale into a binary measure where 1 and 2 are scored as 1 if the item is false and 3 and 4 are scored as 1 if the item is accurate, and then sums these binary ratings (0–18). This excludes respondents who did not complete the full set of ratings (*n*=3). The measures of overconfidence correlate at *r*=0.53.

#### Cancer risk factor (mis)perceptions

We also collected data regarding respondents’ beliefs about cancer risk factors—seven accurate and seven inaccurate statements (Smith et al., 2018). The items are reported in Appendix A.4, see online supplementary material. From these items, we model true and false beliefs separately, as well as a difference score (mean [true items]—mean [false items]).

#### Low-credibility health website visits

We obtained participants’ digital trace data from YouGov’s Pulse Panel, which collects information from individuals who voluntarily opt into online tracking of their digital devices. Our dataset included 9.7 million web addresses across participants, comprising 1.9 million unique URLs and 89,562 distinct parent-level domains. These website visits were recorded over an approximately 4-week period, spanning 2 weeks before and 2 weeks after the survey. We constructed two primary outcome measures for exposure to low-credibility health sites: a binary measure indicating whether a participant visited at least one such domain during the 4-week period and a count measure representing the total number of visits to these sites within this timeframe. A detailed explanation of data processing and coding can be found in Appendix A, see online supplementary material.

#### Additional variables

We collected a number of additional demographic and attitudinal variables. We employ a standard set of preregistered control variables across all primary analyses (age, college, female, non-White); instead of controlling for political variables, we control for personal cancer history as the rating tasks pertain directly to cancer information. See Appendix A.4, see online supplementary material.

### Analytical approach

In our analyses below, we initially provide a series of descriptive statistics about overconfidence. When we move to test RQ2 and RQ3, regarding the potential behavioral correlates of overconfidence, we take a few complementary modeling approaches to account for some limitations of standard techniques. As mentioned above, we construct overconfidence measures, our primary independent variables, as difference scores. This is consistent with a great deal of prior work on behavioral outcomes and focuses on the *gap* between perceived and actual performance, rather than the independent contribution of either (Lyons et al., 2021; Parker & Stone, 2014). However, we go on to isolate the effects of excess perceived ability using two alternate model specifications, given current methodological guidance toward triangulation.

The first, which we preregistered, is a disaggregation approach: we include the constituent terms concurrently in each model. However, this approach does pose some limitations of its own: it ignores the fact that these two components almost certainly influence one another, it is more difficult to interpret given that we are interested in both terms simultaneously, and it assumes constant levels of measurement error across the two terms, when this is likely untrue (Lyons et al., 2021). We additionally employ an exploratory third approach in our online supplementary material (B), again following Lyons et al. (2021). We “residualize” perceived ability, which is a more common approach in current personality and social psychology (Anderson et al., 2012). We fit a regression of perceived ability predicted by actual ability. We then use the estimated residual error as a measure of perceived ability that is unrelated to actual ability.

## Results

We first provide descriptive statistics for overconfidence measures and their components (see online supplementary material, Table S6 and Figure S1).^5^ We begin with the measure of relative overconfidence. Self-rated percentile was above 50 (*t*=13.11, *p*<.001), but only moderately so (*M*=62, median=62), resulting in modest overconfidence, on average: *M*=10.2, median=8 (see online supplementary material, Table S7). A slight majority (57%) showed overconfidence in this measure. Turning to overconfidence (absolute), we found, surprisingly, that the median respondent scored −1 (i.e., estimated they got one fewer correct than they actually did) and a slight majority (52%) were under-confident using this measure (*t*=*−*2.65, *p*=.008). A majority (60%) were within a 3-question margin of error (−3 to 3). Note that we report preregistered analyses of dispositional correlates of overconfidence measures in Appendix C, see online supplementary material.

**Figure 1.**
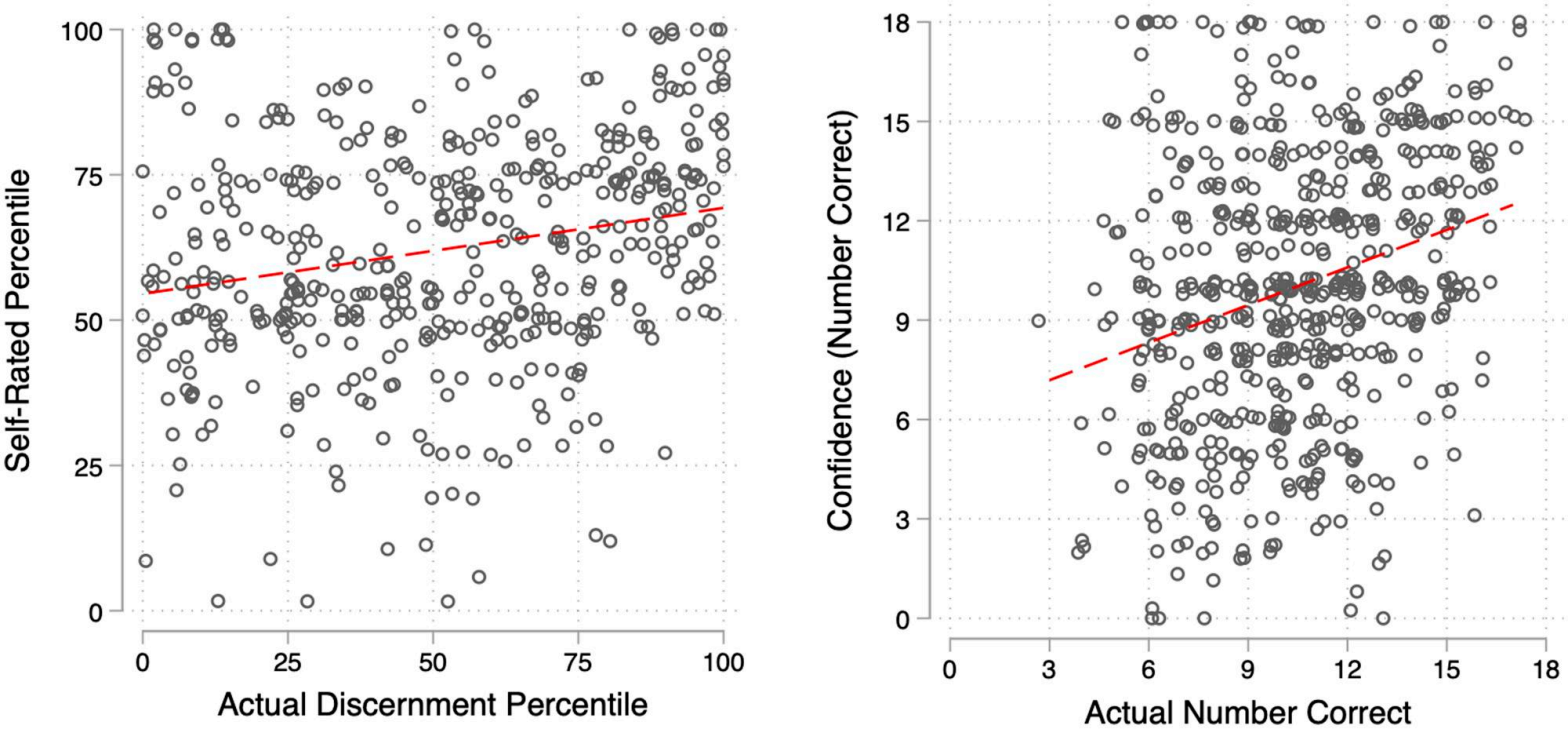
Scatterplots of confidence and actual performance measures. Actual discernment is difference score of performance in distinguishing accurate from inaccurate cancer information, while actual number correct is a sum of correct judgments.

**Figure 2.**
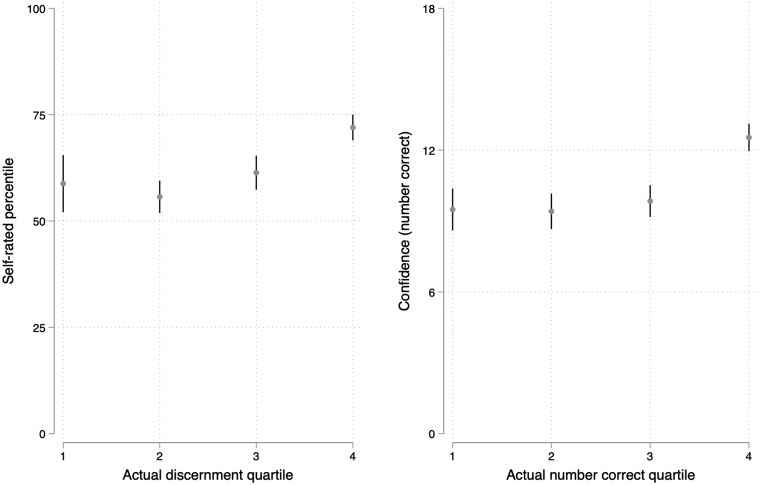
Confidence (relative and absolute) across quartiles of actual performance. Actual discernment is difference score of performance in distinguishing accurate from inaccurate cancer information, while actual number correct is a sum of correct judgments.

We provide scatter plots of the relationship between performance and confidence in Figure 1 (see online supplementary material, Table S8). As expected, those in the lowest quartile of performance did overrate their performance by the greatest degree, exhibiting a classic “Dunning–Kruger” pattern (see online supplementary material, Table S9 and Figure 2): Those in the lowest quartile rated themselves in the 60th percentile, on average, in line with those in the 2nd and 3rd quartiles, while those in the highest quartile of performance rated themselves in the 71st (see equivalent descriptive statistics for absolute measures in Table S10, see online supplementary material).

### Overconfidence is associated with exposure to low-credibility health websites, but this is primarily accounted for by actual performance

**Figure 3.**
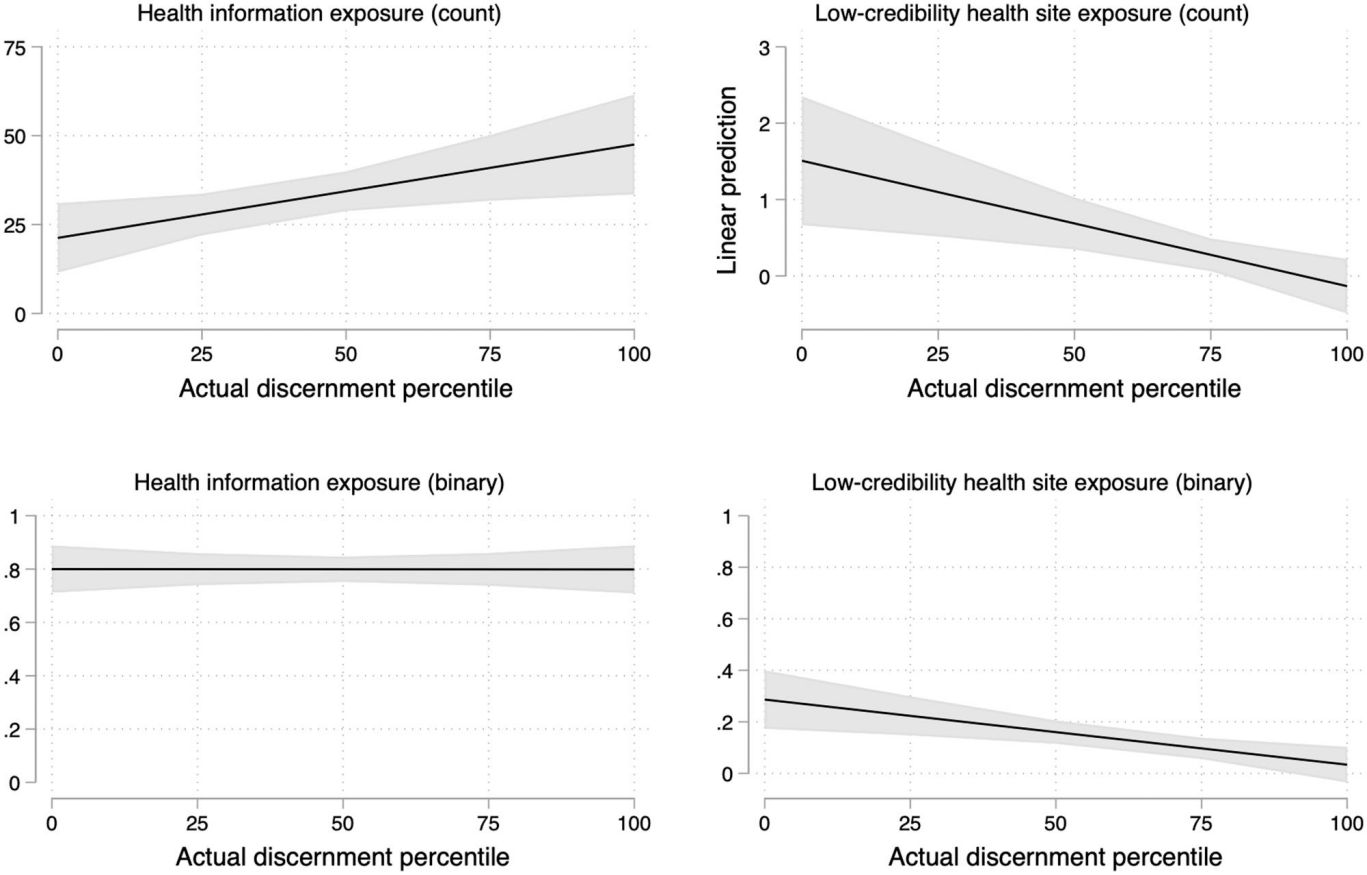
Marginal effects of actual discernment percentile on all health information exposure (left) and exposure to low-credibility health sites (right). Figure shows linear predictions for visits across discernment percentile, drawn from models reported in Tables S11 and S12 (see online supplementary material), which include a set of standard demographic controls and employ survey weights. Actual discernment is a measure of performance in distinguishing accurate from inaccurate cancer information. Note that *y*-axes necessarily differ in range. Browsing data come from a 4-week period of browsing in October–December 2023 among YouGov Pulse panel members.

Next, we model the behavioral correlates of overconfidence using OLS regression with survey weights and a standard set of controls for all models: age, female, college education, non-White racial background, and cancer history. As shown in Table 1, we found that relative overconfidence was associated with greater exposure to low-credibility health sites (binary measure), *b*=.002, *SE*=.001, *p*=.038. In exploratory analysis, we further found that this measure of overconfidence was not associated with general health website exposure (see online supplementary material, Table S11), suggesting the relationship was not due to volume of consumption alone. However, when modeled using the constituent terms (self-rated percentile and actual percentile), we found that the association was primarily due to lower performance in discernment (*b*=*−*.003, *SE*=.001, *p*=.003), rather than the perceptual component. Exploratory follow-up analysis showed that even when tested using separate models, one for each constituent term, self-rated percentile was not associated with exposure (see online supplementary material, Table S12). In additional supplemental analyses, residualized perceived ability was likewise nonsignificant in these models (see online supplementary material, Table S13). Similarly, although the absolute measure of overconfidence was not associated with exposure, the actual number of correct judgments was (Table 1). In Figure 3, we show the marginal effects of actual performance on binary and count measures of exposure to low-credibility health sites, as well as its marginal effects on total health information exposure for comparison.

**Table 1.** Exposure to low-credibility health sites by (over)confidence.

	Exposure (binary)		Exposure (count)	
(a) Relative (over)confidence				

Overconfidence (relative)	0.002*		0.008	
	(0.001)		(0.005)	
Self-rated percentile		−0.001		−0.014
		(0.001)		(0.014)
Actual percentile		−0.003***		−0.016***
		(0.001)		(0.006)
Constant	−0.064	0.122	−0.101	1.770
	(0.043)	(0.088)	(0.171)	(1.130)

Controls	Yes	Yes	Yes	Yes

*R*2	0.12	0.14	0.04	0.07
*N*	407	407	407	407

(b) Absolute (over)confidence				

Overconfidence (absolute)	0.004		0.016	
	(0.004)		(0.030)	
Actual number correct		−0.018*		−0.103**
		(0.007)		(0.039)
Confidence (number correct)		−0.002		−0.022
		(0.004)		(0.035)
Constant	−0.044	0.162*	−0.102	1.217*
	(0.033)	(0.074)	(0.126)	(0.491)

Controls	Yes	Yes	Yes	Yes

*R*2	0.08	0.10	0.04	0.05
*N*	525	525	525	525

*Note*. OLS models with survey weights. Eighteen to twenty-nine years are the reference categories for age. Models include a standard set of covariates: age, female, college education, non-White racial background, and cancer history.

* *p* *<* .05.

** *p* *<* .01.

*** *p* *<* .005 (two-sided).

### Overconfidence is associated with misperceptions about cancer risk factors, but this is primarily accounted for by actual performance

Lastly, we tested whether overconfidence was associated with misperceptions about cancer risk factors at the statement level for false statements, as well as at the respondent level using a difference score for true and false statements, using the same model specifications as above. We found that relative overconfidence was associated with greater belief in false cancer risk factors (*b*=.01, *SE*=.001, *p* *<* .001) and worse discernment between true and false items (*b*=*−*.01, *SE*=.002, *p* *<* .001; Table 2). Decomposing the two terms, again, we found that it was actual performance driving these associations. Again, further exploratory analysis showed that self-rated percentile was not associated with the outcome measures even when employing separate models for each term (see online supplementary material, Table S14). In supplemental analyses, residualized perceived ability was similarly nonsignificant in these models (see online supplementary material, Table S13). As above, we found no association of absolute overconfidence and misperceptions, but a similar pattern of associations for performance in the decomposed models (Table 2).

**Table 2.** Cancer misperceptions by overconfidence.

	False items	True items	False items	True items	Diff. score	Diff. score
(a) Relative (over)confidence						

Overconfidence (relative)	0.007***	−0.003			−0.010***	
	(0.001)	(0.001)			(0.002)	
Self-rated percentile			0.003	0.003		−0.000
			(0.002)	(0.002)		(0.003)
Actual percentile			−0.009***	0.005***		0.013***
			(0.001)	(0.002)		(0.002)
Constant	2.144***	3.141***	2.504***	2.663***	1.127***	0.289
	(0.146)	(0.139)	(0.237)	(0.174)	(0.166)	(0.274)

Controls	Yes	Yes	Yes	Yes	Yes	Yes
Item fixed effects	Yes	Yes	Yes	Yes		

*R*2	0.13	0.09	0.13	0.10	0.20	0.27
*N*	3,241	3,241	3,241	3,241	463	463

(b) Absolute (over)confidence						

Overconfidence (absolute)	0.012	−0.006			−0.019	
	(0.010)	(0.009)			(0.010)	
Confidence (number correct)			0.003	0.011		0.008
			(0.011)	(0.009)		(0.011)
Actual number correct			−0.035*	0.046***		0.080***
			(0.014)	(0.013)		(0.017)
Constant	2.270***	3.084***	2.613***	2.475***	0.940***	−0.012
	(0.136)	(0.132)	(0.227)	(0.174)	(0.159)	(0.243)

Controls	Yes	Yes	Yes	Yes	Yes	Yes
Item fixed effects	Yes	Yes	Yes	Yes		

*R*2	0.09	0.09	0.09	0.10	0.10	0.17
*N*	4,130	4,130	4,130	4,130	590	590

*Note*. OLS models with survey weights. Models include a standard set of covariates: age, female, college education, non-White racial background, and cancer history.

* *p* *<* .05.

*** *p* *<* .005 (two-sided).

### Comparison across studies

Next, we present comparisons with prior work (Lyons et al., 2021), derived from the published work and re-analyses. In Table 3, we show levels of relative (over)confidence in both studies. Broadly, both studies found somewhat similar levels of overconfidence, though there is nuance: Average overconfidence was lower in the current study and a smaller proportion of the sample exhibited overconfidence, but the lowest performers were especially overconfident.^6^

Regarding behavioral correlates, we focused on the most directly comparable outcomes across the studies: binary exposure to untrustworthy websites, and the topical misperception difference score. We do not compare outcomes for the item-level misperception analyses because the Lyons et al. data focused on political misperceptions. These vary in partisan congeniality, which is included in the original study’s models along with an overconfidence *×* congeniality interaction term; there is no direct analog in the cancer misperception analyses. We report a comparison of standardized effect sizes in Table 4 (drawn from models reported in Tables S15 and S16 (see online supplementary material). As shown here, the pattern of associations was fairly similar across the two studies.

Next, we pool the two datasets.^7^ We present main effects of the predictors in the pooled dataset in Table S17 (see online supplementary material). Additionally, we tested for equivalence in these effects across the studies. We used the combined datasets and model the outcomes using a Study variable, with an interaction term for Study and each predictor; the interaction term reflects the difference in the effect between the studies (see online supplementary material, Table S19). The current study showed a significantly stronger negative association between actual ability and exposure compared to Lyons et al. (2021). For the misperception difference score, overconfidence exhibited a significantly stronger negative association in the current study, while actual ability showed a significantly stronger positive association in the current study. Self-rating and residualized self-rating both had relatively weak associations with the misperception difference score in both studies, but these associations were marginally weaker in the current study.

In sum, although we found a similar pattern of associations, we found even stronger support for the primacy of actual performance in predicting both outcomes in the current study.

**Table 3.** Comparison of descriptive results.

	Lyons et al. 1	Lyons et al. 2	Current
	*Politics*	*Politics*	*Health*
Average self-rated percentile	69	69	62
Correlation of self-rating and performance (false items)	0.08	0.10	0.06
Correlation of self-rating and performance (diff. score)^a^	0.22	0.24	0.25
Average overconfidence	22	22	10
Percent of sample overconfident	73%	73%	57%
Bottom quartiles’ average self-rating	63	64	60
Average overconfidence by actual quartile^a^			
Quartile 1	35	35	47
Quartile 2	26	26	19
Quartile 3	16	16	−2
Quartile 4	4	4	−17

a From re-analysis of Lyons et al. (2021).

**Table 4.** Comparison of findings for behavioral correlates (standardized).

Finding	Lyons et al.	Current
	*Politics*	*Health*
Overconfidence *→* binary exposure	0.02 (0.01)**	0.05 (0.03)*
*Perceived ability (controlling for actual)*	0.01 (0.01)	−0.01 (0.03)
*Actual ability (controlling for perceived)*	−0.01 (0.01)*	−0.07 (0.02)***
*Residualized perceived ability*	0.01 (0.01)	−0.01 (0.03)
Overconfidence *→* misperception (diff. score)	−0.13 (0.02)***	−0.38 (0.07)***
*Perceived ability (controlling for actual)*	−0.02 (0.02)	−0.01 (0.07)
*Actual ability (controlling for perceived)*	0.16 (0.02)***	0.48 (0.06)***
*Residualized perceived ability*	−0.04 (0.02)	−0.02 (0.07)

*Note*. For misperception difference score outcomes, entries are standardized betas; outcome and predictor variables are standardized prior to modeling to allow for use of survey weights. For exposure outcomes, we do not standardize the binary measure because of issues associated with doing so. However, we standardize all predictors; coefficients represent change in the outcome for a 1−*SD* change in the predictor. Data come from OLS models reported in Tables S15 and S16 (see online supplementary material). Models include a standard set of covariates and use survey weights.

* *p* *<* .05.

** *p* *<* .01.

*** *p* *<* .005 (two-sided).

## Discussion

Past work shows that most Americans overestimate their ability to discern true from false content online, and this overconfidence is associated with exposure to untrustworthy political outlets and belief in congenial partisan misperceptions. We conducted a conceptual replication and extension of this work, documenting the prevalence and correlates of overconfidence concerning health misinformation. To do so, we rely on a national survey of Americans and linked web-browsing data. Though we find somewhat less overconfidence in the aggregate, the bottom quartile of performers in particular exhibit significantly inflated self-ratings, replicating the DKE in this context. Likewise, the measure of relative overconfidence is associated with exposure to low-credible health websites and worse discernment of accurate and inaccurate cancer risk factors.

However, additional analyses (decomposing constituent terms of overconfidence and residualization of self-ratings) suggest that these associations are likely driven by respondents’ actual discernment underlying our calculation of overconfidence, rather than a lack of metacognitive insight per se. In other words, overconfidence, at least in this domain, is likely a symptom of poor performance, and not a cause of downstream behavioral outcomes. Those holding greater antipathy toward experts may do poorly at discerning credible health information while also consuming more low-credibility content and holding more misperceptions on this topic—regardless of their level of metacognitive awareness (Lyons, 2023). It is important to note here the relative importance of actual performance as noted in the original study as well (Lyons et al., 2021):

"[…] we would assume discernment ability itself—from which our overconfidence measure is in part derived—drives engagement with this content. Unsurprisingly, we find that people who are worse at discerning between legitimate and false news in the context of a survey are worse at doing so in their browsing habits. Further, actual ability is a stronger predictor than perceived ability […] It is not our goal here to argue that overconfidence supersedes ability itself as the key predictor or cause of vulnerability to misinformation, nor do our findings support this interpretation. Indeed, our results lend further support to work that shows ability deficits are a serious issue in this domain."

Overall then, these findings reaffirm the importance of discernment *ability* in predicting deleterious outcomes. Although this was also the case in the original study, equivalence tests suggest that actual performance is even more strongly predictive in the health context of the current study. This finding is directly relevant to the design and deployment of interventions; those targeting skill improvement (Altay, 2022), rather than metacognitive awareness of personal vulnerability, are more likely to improve outcomes in this domain.

The observed domain differences could suggest that overconfidence may emerge from distinct psychological roots depending on context. For politics, inflated self-perceptions often stem from motivated reasoning and identity protection, factors that actively encourage individuals to feel confident in their discernment, often regardless of actual ability. In contrast, overconfidence in the health domain may be more of a byproduct of informational uncertainty, technical complexity, and low task fluency. These features can hinder metacognitive calibration not because individuals are motivated to feel confident, but because the ambiguity of the material makes it harder to accurately gauge one’s performance. As a result, overconfidence in the health context may reflect a passive miscalibration or even a statistical artifact (e.g., regression to the mean among low performers), rather than active self-enhancement. This distinction could help explain why actual ability, not metacognitive miscalibration, emerges as a stronger predictor of behavior in this study.

We also extended the target study by examining an absolute measure of overconfidence alongside a relative measure. Although these were correlated (*r*=.53), there are considerable differences between them. Specifically, we found less overall overconfidence in the absolute measure and essentially no evidence of any dispositional or behavioral correlates. This could suggest that this measure is “noisier” and not a suitable proxy for underlying tendencies and relevant thinking styles, like intellectual humility (Duttle, 2016). Related, our findings regarding the dispositional correlates of relative overconfidence (anti-expert, anti-science, and pro-conspiracist views) may be relevant in applied settings in that interventions could also target these worldviews—sources of underlying distrust and, potentially, cause of poor performance and overplacement on the accuracy rating task—which may be more fruitful than targeting literacy skills alone (Lyons, 2023).

These findings should be interpreted in light of their limitations. Although our conceptual replication extends prior findings to a new domain, we still cannot say whether they generalize more broadly. It is also worth noting ways our design may differ from the target study aside from topic domain. For instance, the difficulty of the headline discernment task may have been more or less difficult, which can influence tendencies toward overplacement and overestimation (Moore & Healy, 2008). Additionally, this study shares certain limitations with the original work it aims to replicate. For instance, the focus is solely on respondents from the United States, our headline set may not fully capture the full range of cancer misinformation in the wild, and domain-level classification of low-credibility visits may miss some misleading content at the page level. Furthermore, because the analyses are correlational, it is important to note that the associations between overconfidence (or actual discernment skill) and the behavioral outcomes explored here should not be interpreted as causal at this time given potential endogeneity concerns. While we focus on individual-level predictors, exposure to low-credibility content likely reflects broader structural and dispositional forces as well (Southwell et al., 2023). From this perspective, misinformation exposure is not merely a behavioral consequence of individual misjudgment, but a phenomenon embedded in deeper systemic and contextual inequalities. Future research might more explicitly examine how individual capacities interact with these structural factors to shape patterns of exposure and belief.

In summary, though we find a link between overconfidence and exposure to low-credibility outlets and belief in relevant misperceptions, follow-up analyses suggest that these relationships are primarily accounted for by (deficits in) actual discernment skill rather than a lack of metacognitive insight. Rather than challenging prior findings that link overconfidence to misinformation engagement, these results may help clarify the boundary conditions of that pattern—suggesting that in domains marked by epistemic uncertainty and weaker identity motivations, overconfidence may arise more as a byproduct of poor performance than from pronounced metacognitive distortions that actively promote risky engagement. Our findings highlight the importance of targeting discernment skills and underlying worldviews in intervention design—particularly in the health domain, where fostering evaluative competence and addressing distrust may prove more effective than simply encouraging metacognitive caution.

## Supplementary Material

hqaf017_Supplementary_Data

## Data Availability

The data underlying this article are available at https://osf.io/nsy87/?view_only=f37575219b56483bba395f45771c94ca.

## Footnotes

1 Differences in measurement approaches may yield less observed overconfidence (Dobbs et al., 2023).

2 Our preregistration is available here: https://osf.io/pd6u8/?view_only=29a2054f79c6439e92faec30b11dc5ba. Data is available at https://osf.io/nsy87/?view_only=f37575219b56483bba395f45771c94ca. A populated pre-analysis plan is also located in Appendix E.

3
Lyons et al. (2021) examine the association of overconfidence and engagement intention. We do not; this requires eliciting both accuracy ratings and engagement intention for each item, which can result in bias (Epstein et al., 2023).

4 Following Lyons et al. (2021), the survey also included an experiment with treatments relating to media literacy reported elsewhere (Lyons et al., 2024). We report tests of these treatments’ effects on overconfidence in Table S23 (see online supplementary material).

5 Following our target study (Lyons et al., 2021), we report unweighted descriptive results but use survey weights in regression analyses below.

6 This modified pattern may reflect several factors; regression to the mean and the better-than-average effect, as noted by recent critiques (Gignac & Zajenkowski, 2020), may play a role, while domain-level factors could also factor in. Cancer misinformation may be more technical or lacking in familiar social cues, making it harder for low-skill individuals to accurately assess their performance. These possibilities are not mutually exclusive.

7 We conduct analyses of the pooled datasets with and without survey weights (normalized across the two studies). We favor the unweighted pooled analyses because they avoid potential biases introduced by normalizing weights (Kish, 1992). However, we report weighted pooled results in Tables S18 and S20 (see online supplementary material).
