# Supplementary material for: Overconfidence in ability to discern cancer misinformation: a conceptual replication and extension
Source: Hum Commun Res. 2025 Jul 14;52(1):66–75. doi: 10.1093/hcr/hqaf017 (PMC12757683; doi:10.1093/hcr/hqaf017)
Supplement: hqaf017_Supplementary_Data [file hqaf017_supplementary_data.zip › SM (v3) formatted.docx]

# Supplementary Materials

Overconfidence in ability to discern cancer misinformation: A conceptual replication and extension

Benjamin Lyons, PhD*1,2,

Andy J. King, PhD1,2

Kimberly A. Kaphingst, ScD1,2

1Department of Communication, University of Utah

Salt Lake City UT, USA

2Cancer Control and Population Sciences, Huntsman Cancer Institute

Salt Lake City UT, USA

*Corresponding Author:

[Ben.lyons@utah.edu](mailto:Ben.lyons@utah.edu)

255 S Central Campus Dr,

Salt Lake City, UT 84112

#

# Supplementary Materials

**Contents**

### [Methods detail](#_bookmark33) A1

- 1. [Sampling and study methodology](#_bookmark34) A1
  2. [Pulse web-tracking data](#_bookmark36) A3
  3. [News headline stimuli](#_bookmark38) A6
  4. [Survey question wording](#_bookmark39) A10
  5. [Summary statistics](#_bookmark40) A12

### [Additional results](#_bookmark41) A13

- 1. [Descriptive results](#_bookmark42) A13
  2. [Behavioral correlates](#_bookmark49) A15
  3. [Comparison with Lyons et al. 2021](#_bookmark54) A17

### [Other preregistered results](#_bookmark61) A22

- 1. [Who is overconfident?](#_bookmark62) A22
  2. [Effects of interventions on overconfidence](#_bookmark67) A26

### [Robustness tests](#_bookmark69) A27

- 1. [Replication using control condition data](#_bookmark70) A27
  2. [Alternative treatment of missing data](#_bookmark80) A32

1. [**Populated pre-analysis plan**](#_bookmark81) **A34**

# Methods detail

## Sampling and study methodology

We draw on data from a survey conducted among a national sample of the U.S. population by the survey company YouGov, which recruits a large panel of opt-in respondents and then uses a weight- ing and matching algorithm to construct a final sample that mirrors the demographic composition of the U.S. population. Data come from a study fielded October 25 - November 21, 2023.

Specifically, YouGov interviewed 1325 respondents who were then matched down to a sample of 1200 to produce the final dataset. The respondents were matched to a sampling frame on gender, age, race, education, and treatment received. The sampling frame is a politically representative "modeled frame" of US adults, based upon the American Community Survey (ACS) public use microdata file, public voter file records, the 2020 Current Population Survey (CPS) Voting and Registration supplements, the 2020 National Election Pool (NEP) exit poll, and the 2020 CES surveys, including demographics and 2020 presidential vote.

The matched cases were weighted to the sampling frame using propensity scores. The matched cases and the frame were combined and a logistic regression was estimated for inclusion in the frame. The propensity score function included age, gender, race/ethnicity, years of education, home ownership status (own, rent, other), 2020 presidential vote, and region. The propensity scores were grouped into deciles of the estimated propensity score in the frame and post-stratified according to these deciles. The weights were then post-stratified on 2020 presidential vote choice, home owner- ship status, region (4-categories), as well as a four-way stratification of gender, age (4-categories), race (4-categories), and education (4-categories), to produce the final weight.

Table S1: Demographic Characteristics of Respondents

**Characteristic Percentage / Value**

| **Gender**  Female | 53% |
| --- | --- |
| Male | 45% |
| Non-binary/Other | 2% |
| **Race/Ethnicity**  White | 71.8% |
| Black | 11.7% |
| Hispanic | 8.8% |
| Asian | 2.8% |
| Native American | 0.8% |
| Two or more races/Other | 4.8% |
| **Age**  Median | 53 |
| Mean (SD) | 51.6 (15.9) |
| Range | 18-89 |

**Education**

Four-year college degree or higher 40%

**Health Insurance**

Had some form of health insurance 91%

**Prior Cancer Diagnosis**

Reported having a prior cancer diagnosis 11.75%

**Household Income**

| Median | $50,000 - $59,999 |
| --- | --- |
| $39,999 or less | 39.9% |
| $40,000 - $79,999 | 33.3% |
| $80,000 - $119,999 | 12.4% |
| Over $120,000 | 14.1% |

## Pulse web-tracking data

**Summary** A more detailed explanation of data processing and coding can be below, but we sum- marize key aspects here. Initially, URLs were categorized for health-related content (1 = pres- ence of health information). This classification was based on the "Health" category tag automati- cally assigned by the data provider, supplemented by an additional identification process to capture any relevant URLs that may have been missed. Specifically, URLs labeled as "News and Media" or "Forums" were further evaluated using the OpenAI “text-embedding-3-small” model via the Python OpenAI API, and manually inspected. Next, within domains tagged as "health," we man- ually coded a "low-credibility" health domain label. This was a binary classification indicating whether a domain was considered credible (0) or not (1), with two coders independently assessing all sites. The manual coding occurred at the level of the domain. Among the 1,155 health domains, only 78 (6.8%) were determined to be low-credibility. Key criteria for this classification included sites containing inaccurate or misleading health information, those primarily focused on selling products with unverified claims, sites flagged as "deceptive" or "untrustworthy" by web browsers, and those lacking credible sources, external links, or exhibiting characteristics of large language model-generated content. The two coders demonstrated complete agreement in their credibility assessments (Krippendorff’s alpha = 1.00).

Based on this classification, we constructed two primary outcome measures for exposure to low- credibility health sites: a binary measure indicating whether a participant visited at least one such domain during the four-week period and a count measure representing the total number of visits to these sites within this timeframe.

### Data preparation documentation

The final labeled dataset has 9,940,481 rows and 78 columns with a total of 1,059 respondents.

To prepare the data for analysis, we created the following binary variables.

### Category

The original dataset had a pre-coded “category” tag column, which included text tags such as “News and Media”, “Health”, “Shopping”, “Forums”, etc. These variables correspond to vari- ables for each category tag. For example, the variable “category shopping” equals 1 if the original “category” variable contained the “Shopping” tag and is equal to 0 otherwise. Likewise for all 44 category tags. Note that a URL can have multiple category tags. When the pre-coded “Health” category tag was evaluated using a random sample of 500 URLs that were human-coded, the tags had a true positive rate of 0.95, a true negative rate of 0.95, and a Cohen’s Kappa score of 0.89.

### News (health)

Since the “category” variable does not always capture news/media and forum URLs that are health-related (such as health-related news articles and reddit posts), we used a text-embedding approach to label all URLs with a “News and Media” or “Forums” as health-related or not. The “news health” variable, which specifies whether each URL with a “News and Media” or “Forums” tag is health-related or not, is computed using a text-embedding model.

Specifically, we used the OpenAI “text-embedding-3-small” model via the Python OpenAI API to generate 1,536-dimensional text embeddings for each of the 75,062 unique URLs with a “News and Media” or “Forums” tag. To create a “news health” label for each of these URLs, we computed the cosine distance from the URL’s embedding to both a “health” reference embedding and a “non- health” reference embedding. The “health” news reference embedding is computed based on the

average embedding position of 55 news/forum URLs that were manually determined (i.e., human- coded) to be health related, and similarly, the “non-health” news reference embedding is computed based on the average embedding position of 116 news/forum URLs that were manually determined (i.e., human-coded) to be non-health related. These averages were computed across each of the 1,536 dimensions in the reference URL embeddings.

If a URL’s embedding is closer (in terms of cosine distance) to the average “health” reference embedding, the “news health” variable is given value 1, else the average “news health” variable is given value 0.

The 55 health and 116 non-health reference URLs were chosen by looking at random samples of the URLs and manually identifying health-related URLs and non-health-related URLs, with a focus on sequentially including URLs that were being misclassified by the embedding labeling model described here to improve the algorithmic accuracy. Note that this model identifies 2,757 news-related health URLs, which accounts for only 6% of all health-related URLs (based on the health category tag + the news/forums health URLs).

To validate the automated classification of URLs as health-related, we conducted a manual coding task on a stratified sample of 300 URLs. This included 100 URLs coded by the algorithm as health-related, 100 News and Media URLs coded as non-health-related, and 100 Fourm URLs coded as non-health-related. Two human coders evaluated each URL for health-related content. Among the health-related news/forum URLs, 97% were confirmed by human coders (true positive rate). Among the non-health-coded URLs, true negative rates were 97% for Forums and 100% for News and Media URLs. Overall, inter-rater agreement between human and automated coding was strong, with a Cohen’s kappa score of 0.96.

### Health

This variable specifies whether each URL is health-related or not. This variable is equal to 1 if either the URL has a health “category” tag in the original data, or if the “news health” variable (defined above) is equal to 1. Otherwise, the variable has value 0. There are a total of 45,395 URLs with a “health” value of 1 (compared with 9,895,086 URLs with a “health” value of 0).

### Low-credibility domain

A “low-credibility” label was hand-coded by two coders for all URLs that had a “health” tag in the original dataset based on whether the URL was deemed to be credible (0) or not (1), with both coding all sites. This hand-coding was based only on the domain portion of the URL. Of the 1,155 domains with a health category tag, only 78 (6.8%) were manually determined to be low- credibility. Primary criteria were site including inaccurate or misleading health information, site focusing primarily or heavily on sales products, site indicated as “deceptive,” “untrustworthy,” or similar by web browser, or site featuring content that primarily lacked sources, links to credible sites, or appeared to be generated by large language models. Our two coders exhibited agreement on credibility ratings (Krippendorf’s alpha = 1.00).

We additionally created a sub-code for all low-credibility domains called "Sales." This sub-code indicates if the domain appears to exist primarily to sell health products, rather than disseminate news, information, or other content. Our two coders also reached high agreement for this sub-code (Krippendorf’s alpha = .92). We denote all sales domains in Table [S2](#_bookmark37) with an asterisk.

Table S2: Low-credibility health domains

7minutewealthmagnet.com* alpilean.com*

curezone.org drpsychological.com exoduseffect.com* getneuropure.com* guthealthwellness.com* healthandwellnesstools.com hsicures.com lifeextension.com* longevityactivation.com* naturalhealthreports.net* nervepaindiscovery.com* parasiterelief.com* peoplespharmacy.com reitschuster.de sanesolution.com* simplyhealth.io solairehealth.org teambeachbody.com* thehealthyfat.com* trysightcarefast.org*

trusted-10.com warningerchiropractic.com

actionmatters.org childrenshealthdefense.org dailyom.com* earthclinic.com* expertsinhealth.co* gundrymd.com*

healthy-guru.com* healthnwell.com hsionlineorders.net lifedailytrends.com* mercola.com naturalhealthresponse.com nutritionandhealing.com perfectbody.me* puravive.com* remedydaily.com scarysymptoms.com* sleepcream.com* spiritualityhealth.com theayurvedaexperience.com* thenewgutfix.com* tryarmra.com* truthaboutabs.com* whydoctorslie.com

agelessbrainformula.com* covid19criticalcare.com drberg.com

emuaid.com* findpronto.com gutcleanseprotocol.com* healthy-holistic-living.com healthydirections.com* hylands.com* liverrenew.com* metaboosting.com* naturemdonline.com* organicolivia.com* perfectorigins.com* qualityhealth.com

rmbl.ws simplebloodsugarfix.com* sleepcreme.com* standardprocess.com* thegutrehab.com* thetappingsolution.com* tryfitspresso.org* upwellness.com*

y-ourskin.com*

Low-credibility sales domains indicated by *

## News headline stimuli

Our aim was to sample headlines that reflect the kinds of claims individuals are likely to encounter online, particularly on social media and popular news sources. In this, we follow the target study in using a curated headline set. To qualify as stimuli, all articles needed a URL in order to create social media headline previews and generally needed to present a factual claim. To ensure topical coherence, all headlines were drawn from cancer-related content. This focus allowed us to stan- dardize content difficulty and relevance, while still capturing individual differences in evaluative accuracy that generalize to broader health misinformation exposure.

To construct the headline set, we drew inaccurate items from fact-checking databases such as Snopes or PolitiFact Health with claims about cancer flagged as false or misleading. In addition to these fact-checks, we supplemented with high-visibility cancer misinformation presented when web searching common cancer keywords. For accurate headlines, we used Google News – Health to locate recent mainstream news about cancer. These headlines are drawn from major national health journalism outlets, universities, and similar sources. In both cases, we prioritized headlines that were timely and/or reflected recurrent themes in cancer (mis)information. For both accurate and inaccurate categories, veracity was further vetted by cancer experts at [blinded]. Notably, the head- line stimuli each respondent rated were sampled from a large pool to increase diversity. Respon- dents rated 6 inaccurate headlines (randomly drawn from a pool of 16) and 12 accurate headlines (randomly drawn from a pool of 44).

Importantly, our aim was not to capture a headline set fully representative of all cancer news; our confidence questions specified that respondents were to rate their discernment performance in the present study only. While our set is not a probabilistic sample of all health headlines, we followed the curated approach of the target study and common guidance in the misinformation literature (Pennycook et al., [2021](#_bookmark89)), in which scholars typically use curated sets of verified true/false headlines that aim to simulate realistic exposure rather than exhaustive population coverage (though see Aslett et al. ([2024](#_bookmark82)) for a recent attempt to create algorithmically selected representative selections).

The order of the headlines was randomized for each respondent. Headlines were presented as they would appear on the Facebook News Feed to enhance ecological validity (featuring a headline, photo, and the news source’s web domain). The full set of headline images used the study can be accessed here: <https://osf.io/nsy87/?view_only=f37575219b56483bba395f45771c94ca>

## Inaccurate headlines and URLs

Top Scientists Find ‘Substantial Scientific Evidence’ RF Radiation Causes Cancer <https://childrenshealthdefense.org/defender/radiofrequency-rf-radiation-cancer/>

Top Pathologist confirms Cancer, Infertility & Strange Blood Clots are common side effects of Covid-19 Vaccination <https://expose-news.com/2023/04/22/cancer-infertility-blood-clots-due-to-covid-vaccination/>

The COVID-19 mRNA “Vaccines” cause Cancer; here’s the evidence. . . <https://expose-news.com/2022/08/02/the-covid-19-mrna-vaccines-cause-cancer/>

Are COVID Boosters Behind Increase in Cancer Among Younger Adults? <https://childrenshealthdefense.org/defender/covid-boosters-increase-cancer-young-adults-cola/>

THIS SMALL FRUIT IS A REAL TREASURE: CAN CURE PROSTATE CANCER AND PREVENT STOMACH AND COLON CANCER!

<https://instiks.com/this-small-fruit-is-a-real-treasure-can-cure-prostate-cancer-and-prevent-stomach-and-colon-cancer/>

50-Year-Old Man Cures Lung Cancer With Cannabis Oil, Stuns CBS News

[http://4healthmm.com/50-year-old-man-cures-lung-cancer-cannabis-oil-stuns-cbs-news/#:~:tex$t=$4%20Health%20MM-,50%2DYear%2DOl](http://4healthmm.com/50-year-old-man-cures-lung-cancer-cannabis-oil-stuns-cbs-news/#%3A~%3Atex%24t%20%3D%20%244%20Health%20MM-%2C50%2DYear%2DOld%20Man%20Cures%20Lung%20Cancer%20With%2CCannabis%20Oil%2C%20Stuns%20CBS%20News%26tex%24t%20%3D%20%24A%20combination%20of%20cannabis%20oil%2Cothers%20heal%20their%20lives%2C%20too)

[d%20Man%20Cures%20Lung%20Cancer%20With,Cannabis%20Oil%2C%20Stuns%20CBS%20News&tex$t=$A%20combination%20of%20](http://4healthmm.com/50-year-old-man-cures-lung-cancer-cannabis-oil-stuns-cbs-news/#%3A~%3Atex%24t%20%3D%20%244%20Health%20MM-%2C50%2DYear%2DOld%20Man%20Cures%20Lung%20Cancer%20With%2CCannabis%20Oil%2C%20Stuns%20CBS%20News%26tex%24t%20%3D%20%24A%20combination%20of%20cannabis%20oil%2Cothers%20heal%20their%20lives%2C%20too) [cannabis%20oil,others%20heal%20their%20lives%2C%20too.](http://4healthmm.com/50-year-old-man-cures-lung-cancer-cannabis-oil-stuns-cbs-news/#%3A~%3Atex%24t%20%3D%20%244%20Health%20MM-%2C50%2DYear%2DOld%20Man%20Cures%20Lung%20Cancer%20With%2CCannabis%20Oil%2C%20Stuns%20CBS%20News%26tex%24t%20%3D%20%24A%20combination%20of%20cannabis%20oil%2Cothers%20heal%20their%20lives%2C%20too)

Kashmiri scientist discovers prostate cancer treatment

<https://www.greaterkashmir.com/todays-paper/kashmiri-scientist-discovers-prostate-cancer-treatment>

Cow urine cured my breast cancer: Sadhvi Pragya

<https://www.indiatoday.in/elections/lok-sabha-2019/story/sadhvi-pragya-cow-urine-cancer-1507816-2019-04-22>

Women recently injected with experimental covid vaccines are showing symptoms of BREAST CANCER <https://dcdirtylaundry.com/women-recently-injected-with-experimental-covid-vaccines-are-showing-symptoms-of-breast-cancer/>

Cancer Is a Fungus, Cancer Is a Frequency: Dr. Darrell Wolfe <https://thesternmethod.com/darrell-wolfe/>

The Cancer Industry is too prosperous to allow a cure <https://www.naturalnews.com/055697_cancer_industry_big_pharma_natural_cures.html>

Cover-Up Of Promising Cancer Treatment | Cancer Research | Documentary <https://www.youtube.com/watch?v=ee9KCGZvVfA>

Top 7 reasons chemotherapy fails more than 97% of the time, creating new cancers in the body and crippling the chances of true recovery <https://www.naturalnews.com/054324_cancer_chemotherapy_natural_cures.html>

The Sugar And Cancer Connection <https://natureworksbest.com/sugar-feeds-cancer-growth/>

Can cancer really be cured with ivermectin and other safe, old treatments? <https://drtesslawrie.substack.com/p/can-cancer-really-be-cured-with-ivermectin/comments>

Man With “Terminal” Lung Cancer Cures Himself With Cannabis Oil <https://www.thehealthcure.org/blog/man-with-terminal-lung-cancer-cures-himself-with-cannabis-oil/>

## Accurate headlines and URLs

Video distraction helps kids undergo cancer radiotherapy, Stanford Medicine-led study finds <https://med.stanford.edu/news/all-news/2023/05/kids-cancer-video-distraction.html>

Gene-editing technique could speed up study of cancer mutations <https://news.mit.edu/2023/gene-editing-technique-cancer-mutations-0511>

Antibiotics after breast cancer linked to poorer survival, Stanford Medicine-led study finds <https://med.stanford.edu/news/all-news/2023/04/antibiotics-cancer.html>

Breast density changes over time could be linked to breast cancer risk, study finds <https://www.cnn.com/2023/05/02/health/breast-density-cancer-study/index.html>

Vaping may ‘wake up’ cancer cells and trigger wave of disease in a decade

<https://www.independent.co.uk/life-style/health-and-families/health-news/vaping-ecigarettes-cancer-research-b2165228.html>

Very few Americans know drinking alcohol increases cancer risk, study finds <https://www.today.com/health/doctors-sound-alarm-alcohol-cancer-link-study-finds-americans-aware-rcna59696>

Research shows promising results for future of cancer treatments through vaccines <https://abcnews.go.com/Health/video/research-shows-promising-results-future-cancer-treatments-vaccines-99254988>

You don’t need to walk 10,000 steps a day — walking faster is what counts to protect you from heart disease and cancer <https://news.yahoo.com/dont-walk-10-000-steps-170514582.html>

Health panel recommends women get screening mammograms at age 40 <https://www.washingtonpost.com/wellness/2023/05/09/mammogram-age-40-breast-cancer-screening/>

A Scary New Link Between Air Pollution and Lung Cancer

[https://www.washingtonpost.com/business/a-scary-new-link-between-air-pollution-and-lung-cancer/2022/09/15/91654f22-350f-11ed-a0d6-4](https://www.washingtonpost.com/business/a-scary-new-link-between-air-pollution-and-lung-cancer/2022/09/15/91654f22-350f-11ed-a0d6-415299bfebd5_story.html) [15299bfebd5_story.html](https://www.washingtonpost.com/business/a-scary-new-link-between-air-pollution-and-lung-cancer/2022/09/15/91654f22-350f-11ed-a0d6-415299bfebd5_story.html)

Link Between Long Telomeres and Long Life Is a Tall Tale, Study Finds <https://www.nytimes.com/2023/05/04/health/long-telomeres-age-longevity.html>

Sugary drinks associated with increased risk of death from cancer

<https://www.msn.com/en-us/health/medical/sugary-drinks-associated-with-increased-risk-of-death-from-cancer/ar-AA11RXmm>

Genetic test for cancer is less accurate for Black and Asian people

<https://www.msn.com/en-us/health/medical/genetic-test-for-cancer-is-less-accurate-for-black-and-asian-people/ar-AA12peqj>

New mRNA Pancreatic Cancer Vaccine Trial Starts Next Phase

<https://www.mskcc.org/news/can-mrna-vaccines-fight-pancreatic-cancer-msk-clinical-researchers-are-trying-find-out>

For Advanced Endometrial Cancer, Chemotherapy Plus Immunotherapy Improves Outcomes <https://www.mskcc.org/news/advanced-endometrial-cancer-chemotherapy-plus-immunotherapy-improves-outcomes>

Screen all women for breast cancer at 40, instead of 50, new guidelines say <https://www.nbcnews.com/health/womens-health/breast-cancer-guidelines-start-screenings-age-40-rcna83355>

Pancreatic Cancer Vaccine Shows Promise in Small Trial <https://www.nytimes.com/2023/05/10/health/pancreatic-cancer-vaccine-mrna.html>

An mRNA vaccine that programs the body to fight pancreatic cancer shows early promise <https://www.cnn.com/2023/05/10/health/pancreatic-cancer-vaccine/index.html>

Rare Melanoma Very Likely to Respond to Treatment with Pembrolizumab

<https://www.cancer.gov/news-events/cancer-currents-blog/2023/pembrolizumab-alone-desmoplastic-melanoma>

Too Many Older Men Are Still Screened for Prostate Cancer

<https://www.nytimes.com/2023/05/08/health/prostate-cancer-screening.html?auth=login-email&login=email&searchResultPosition=1>

AI vs. cancer: AstraZeneca exec reveals how COVID pandemic helped develop early cancer diagnosis tech <https://www.foxnews.com/media/ai-cancer-astrazeneca-exec-covid-pandemic-helped-tech-early-cancer-diagnosis>

Cancer Research Points to Key Unknowns about Popular “Antiaging” Supplements <https://www.scientificamerican.com/article/cancer-research-points-to-key-unknowns-about-popular-antiaging-supplements/>

Transgender women on hormones face higher risk of prostate cancer going undiagnosed: Study <https://www.foxnews.com/media/transgender-women-hormones-higher-risk-prostate-cancer-undiagnosed-study>

Dermatologists found the ’world’s smallest skin cancer’ under a woman’s eye — it was smaller than the tip of a pen

[https://www.msn.com/en-us/health/medical/dermatologists-found-the-world-s-smallest-skin-cancer-under-a-woman-s-eye-it-was-smaller-tha](https://www.msn.com/en-us/health/medical/dermatologists-found-the-world-s-smallest-skin-cancer-under-a-woman-s-eye-it-was-smaller-than-the-tip-of-a-pen/ar-AA1aHaVm) [n-the-tip-of-a-pen/ar-AA1aHaVm](https://www.msn.com/en-us/health/medical/dermatologists-found-the-world-s-smallest-skin-cancer-under-a-woman-s-eye-it-was-smaller-than-the-tip-of-a-pen/ar-AA1aHaVm)

Artificial intelligence helping detect early signs of breast cancer in some US hospitals

[https://www.msn.com/en-us/health/medical/artificial-intelligence-helping-detect-early-signs-of-breast-cancer-in-some-us-hospitals/ar-AA1aF](https://www.msn.com/en-us/health/medical/artificial-intelligence-helping-detect-early-signs-of-breast-cancer-in-some-us-hospitals/ar-AA1aFDMU) [DMU](https://www.msn.com/en-us/health/medical/artificial-intelligence-helping-detect-early-signs-of-breast-cancer-in-some-us-hospitals/ar-AA1aFDMU)

Inhaled steroids for asthma may offer some cancer protection, study says <https://www.washingtonpost.com/wellness/2023/05/01/asthma-cancer-steroids/>

Change in Oral Sex Attitudes Causing Rise in HPV-Related Throat Cancer <https://www.newsweek.com/oral-sex-hpv-infections-increasing-throat-cancer-1797374>

Love that ‘new car smell’? Study says there are cancer-causing chemicals to consider <https://www.foxnews.com/health/love-new-car-smell-study-says-there-are-cancer-causing-chemicals-consider>

Blood-test biopsy could speed up cancer treatment

<https://www.msn.com/en-xl/news/other/blood-test-biopsy-could-speed-up-cancer-treatment/ar-AA1aiETu>

Cancer survivors may be at heightened risk of cardiovascular disease <https://www.theguardian.com/society/2023/apr/18/cancer-survivors-heightened-risk-heart-cardiovascular-disease>

Liquid Biopsies on the Horizon for Children with Solid Cancer

<https://www.cancer.gov/news-events/cancer-currents-blog/2023/liquid-biopsy-children-solid-cancers>

EPA proposes first-ever standard for cancer causing chemicals in drinking water <https://www.foxnews.com/politics/epa-proposes-first-ever-limit-cancer-causing-chemicals-drinking-water>

Artificial turf potentially linked to cancer deaths of six Phillies ball players – report <https://www.theguardian.com/society/2023/mar/10/phillies-ball-players-cancer-artifical-turf>

Cancer will cost the world $25 trillion over next 30 years <https://www.nature.com/articles/d41586-023-00634-9>

After 30 years of research, pill developed for breast cancer approved for use

<https://www.seattletimes.com/seattle-news/health/after-30-years-of-research-pill-developed-for-breast-cancer-approved-for-use/>

An ant’s sense of smell is so strong, it can sniff out cancer <https://www.washingtonpost.com/climate-environment/2023/01/24/ants-smell-cancer/>

Child cancer rates higher than normal in New Jersey town with contaminated water <https://www.foxnews.com/us/child-cancer-rates-higher-normal-new-jersey-town-contaminated-water>

Cancer Vaccine Created via CRISPR Prevents and Stops Brain Tumors

<https://www.psychologytoday.com/us/blog/the-future-brain/202301/cancer-vaccine-created-via-crispr-prevents-and-stops-brain-tumors>

’Overwhelming’ response for radon testing after Lehi woman shares cancer diagnosis story <https://www.ksl.com/article/50561517/overwhelming-response-for-radon-testing-after-lehi-woman-shares-cancer-diagnosis-story>

Patients with cancer at greater risk of suicide <https://www.upi.com/Health_News/2023/01/23/cancer-diagnosis-suicide-risk/2281674231882/>

Pancreatic Cancer Vaccine Shows Promise in Small Initial Trial <https://www.scientificamerican.com/article/pancreatic-cancer-vaccine-shows-promise-in-small-initial-trial/>

Safest sunscreens to use this summer, according to experts <https://www.cnn.com/2023/05/23/health/sunscreen-guide-2023-wellness/index.html>

"Radioactive" roads made of potentially cancer-causing mining waste could be headed to Florida under new bill <https://www.cbsnews.com/news/radioactive-roads-phosphogypsum-mining-could-be-headed-to-florida/>

Cancer Vaccines Poised to Unlock ’New Treatment Paradigm’ With Merck/Moderna Data

[https://www.usnews.com/news/top-news/articles/2023-06-05/cancer-vaccines-poised-to-unlock-new-treatment-paradigm-with-merck-moderna](https://www.usnews.com/news/top-news/articles/2023-06-05/cancer-vaccines-poised-to-unlock-new-treatment-paradigm-with-merck-moderna-data)

[-data](https://www.usnews.com/news/top-news/articles/2023-06-05/cancer-vaccines-poised-to-unlock-new-treatment-paradigm-with-merck-moderna-data)

## Survey question wording

Note: We use demographic measures as provided by YouGov.

**Health insurance**

Do you currently have health insurance?

- - - Yes, I have private insurance (for example, through my job, HMO)
    - Yes, I have public insurance (for example, Medicaid, Medicare)
    - No

**Binary cancer history indicator**

Have you ever been diagnosed with cancer?

- - - Yes
    - No
    - [If yes] What type? [open]

**Anti-expert views**

Average of 3 items, 5 pt Likert (agree/disagree):

- - - I am more confident in my opinion than other people’s facts
    - Most of the time I know just as much as experts
    - Experts really don’t know that much

**Conspiracism**

Average of 4 items, 5 pt Likert (agree/disagree):

- - - Much of our lives are being controlled by plots hatched in secret places.
    - Even though we live in a democracy, a few people will always run things anyway.
    - The people who really ’run’ the country are not known to the voter.
    - Big events like wars, recessions, and the outcomes of elections are controlled by small groups of people who are working in secret against the rest of us.

**“Low-end” digital literacy scale**

Average of 5 items, (Never/Almost never/Occasionally/Somewhat often/Very often):

- - - I rely on family members to introduce me to new technology.
    - I have professionals (such as the Geek Squad) or family members take a look at my computer when something isn’t working.
    - A lot of the things I see online confuse me.
    - I have problems with viruses and malware on my computer.
    - I have trouble finding things that I’ve saved on my computer.

**Brief Health Literacy Screener**

Average of 4 items

(Always, Often, Sometimes, Occasionally, Never):

- - - How often do you have someone help you read hospital materials?
    - How often do you have problems learning about your medical condition because of difficulty understanding written information?
    - How often do you have a problem understanding what is told to you about your medical condition? (Not at all, A little bit, Somewhat, Quite a bit, Extremely):
    - How confident are you filling out medical forms by yourself?

*Note: Regarding these scaled items, we employ a “low-end” digital literacy scale developed to separate low-skill users from the rest of the population (A. M. Guess & Munger, [2023](#_bookmark83)), where high scores indicate more self-reported diffi- culty with tasks relating to online information (4 items, Cronbach’s alpha = .64), and the Brief Health Literacy Screen (BHLS), standard measure of subjective health literacy (Wallston et al., [2014](#_bookmark90)) (4 items, Cronbach’s alpha = .77). Fur-

ther, we use previously validated scales for anti-expert sentiment (3 items, Cronbach’s alpha = .75) and conspiracism (4 items, Cronbach’s alpha = .87) (Han et al., [2022](#_bookmark85)).

**Feeling Thermometers**

(1-100 sliders)

Next, we would like to know your feelings toward some groups of people using something we call the feeling ther- mometer. Ratings between 50° and 100° mean that you feel favorable and warm toward the person or the group of people. Ratings between 1° and 50° mean that you don’t feel favorable toward the person or group of people and that you don’t care too much for them. You would rate the person or group of people at the 50° mark if you don’t feel particularly warm or cold toward them.

- - - Pharmaceutical companies
    - Doctors
    - News media
    - Scientists in general
    - Social media

**Cancer Perceptions Battery (note: measured pre-treatment)**

5 pt Likert (agree/disagree, “not sure” as the midpoint).

How much do you agree that each of these can increase a person’s chance of developing cancer?

- - - Exposure to non-ionizing electromagnetic frequencies (WiFi, 5G, radio)*
    - Feeling stressed*
    - Using cleaning products*
    - Eating genetically modified food*
    - Getting a COVID-19 vaccine*
    - Using microwave ovens*
    - Drinking from plastic bottles*
    - Drinking more than 1 unit of alcohol a day
    - Being overweight or obese (BMI over 25)
    - Getting sunburnt more than once as a child
    - Being over 70 years old
    - Having a close relative with cancer
    - Infection with HPV (human papillomavirus)
    - Doing less than 30 minutes of physical activity 5 times a week

## Outcome measures

**Perceived accuracy**

4 pt. (“Not at all accurate” (1) to “Very accurate” (4))

To the best of your knowledge, how accurate is the claim in the above headline?

**Confidence**

How do you think you compare to other Americans in how well you performed in this study at recognizing news that is made up? Please respond using the scale below, where 1 means you’re at the very bottom (worse than 99% of people) and 100 means you’re at the very top (better than 99% of people). [1-100 slider]

You just made 18 news evaluations. How many do you think you got right? [0-18]

## Summary statistics

Table S3: Cancer risk factor belief acceptance (unweighted)

|  | **Proportion (SEs)** |
| --- | --- |
| **Inaccurate Cancer Risk Factor Beliefs** |  |
| Exposure to non-ionizing electromagnetic frequencies (WiFi, TV, radio) | 0.158 (0.011) |
| Feeling stressed | 0.360 (0.014) |
| Using cleaning products | 0.296 (0.013) |
| Eating genetically modified food | 0.158 (0.011) |
| Getting a COVID-19 vaccine | 0.122 (0.009) |
| Using microwave ovens | 0.141 (0.010) |
| Drinking from plastic bottles | 0.182 (0.011) |
| **Accurate Cancer Risk Factor Beliefs** |  |
| Drinking more than 1 serving of alcohol a day | 0.323 (0.013) |
| Being overweight or obese (BMI over 25) | 0.478 (0.014) |
| Getting sunburnt more than once as a child | 0.558 (0.014) |
| Being over 70 years old | 0.425 (0.014) |
| Having a close relative with cancer | 0.726 (0.013) |
| Infection with HPV (human papillomavirus) | 0.552 (0.014) |
| Doing less than 30 min of physical activity 5 times a week | 0.293 (0.013) |

Table S4: Descriptive statistics for headline ratings and cancer risk factor beliefs (unweighted)

| **Variable** | **N** | **M** | **SD** | **Min** | **Max** |
| --- | --- | --- | --- | --- | --- |
| Headline discernment | 593 | 0.69 | 0.72 | -1.33 | 2.75 |
| Perc. accuracy (inaccurate) | 593 | 1.86 | 0.64 | 1 | 4 |
| Cancer misperception (diff. score) | 1,200 | 0.65 | 0.82 | -2.43 | 3.29 |
| Cancer misperception (false) | 1,200 | 2.67 | 0.73 | 1 | 5 |

Note that headline discernment is computed as mean (accurate news accuracy) - mean (inaccurate news accuracy); cancer risk misperception (diff. score) is computed as mean (true items) - mean (false items).

Table S5: Descriptive statistics for health exposure trace data (unweighted)

| **Variable** | **N** | **M** | **SD** | **Min** | **Max** |
| --- | --- | --- | --- | --- | --- |
| Low-credibility health site exposure (binary) | 1,059 | 0.14 | 0.35 | 0 | 1 |
| Total low-credibility visits | 1,059 | 0.65 | 4.50 | 0 | 122 |
| Health information exposure (binary) | 1,059 | 0.80 | 0.40 | 0 | 1 |
| Total health visits | 1,059 | 42.86 | 93.06 | 0 | 1064 |

Data come from a four week period in October-December 2023 among YouGov Pulse panel members (N = 1,059).

# Additional results

## Descriptive results


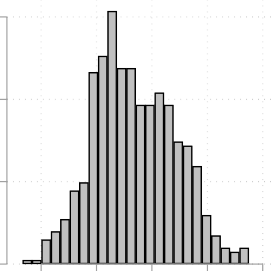
.6

.4

Density

.2

0

-1 0 1 2 3

Actual discernment

.03

.02

Density

.01

0


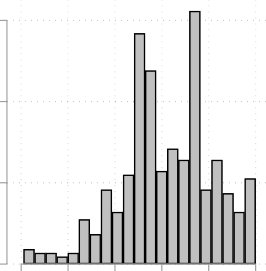


0 20 40 60 80 100

Self-rated percentile

.015

.01

Density

.005

0


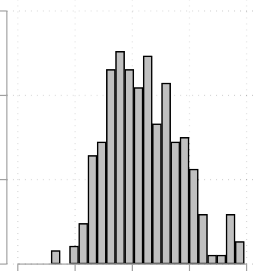


-100 -50 0 50 100

Overconfidence (relative)

.15

.1

Density

.05

0


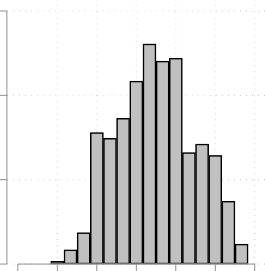


0 3 6 9 12 15 18

Actual number correct

.15

.1

Density

.05

0


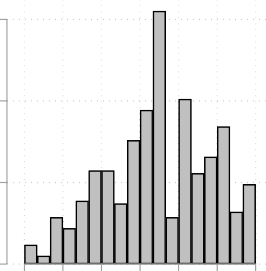


0 3 6 9 12 15 18

Confidence (number correct)

.15

.1

Density

.05

0


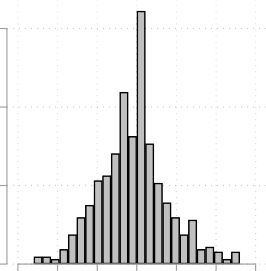


-15 -10 -5 0 5 10 15

Overconfidence (absolute)

### Figure S1: Histograms of performance and (over)confidence measurements.

Table S6: Unweighted summary statistics for outcomes

| Variable | *M* | *SD* | Min | Max |
| --- | --- | --- | --- | --- |
| Self-rated percentile | 62.23 | 20.06 | 1 | 100 |
| Confidence (number correct) | 10.00 | 4.15 | 0 | 18 |
| Actual number correct | 10.50 | 2.98 | 3 | 17 |
| Overconfidence (relative) | 10.18 | 31.72 | -71 | 98 |
| Overconfidence (absolute) | -0.48 | 4.38 | -13 | 13 |

The (unweighted) correlation of discernment and self-rated percentile was *r* = 0.24. The cor- relation of actual number correct and confidence (number correct) was *r* = 0.27.8 We conducted OLS regressions assessing these associations while adding covariates: age, sex, college education, nonwhite racial background, and cancer history (note that all OLS models use survey weights, and leave percentile-based measures in their natural scale). In both cases, actual performance strongly predicted confidence (Table [S8](#_bookmark46)).

Table S7: Overconfidence *t*-tests

|  | **Mean (SE)** | **95% CI** | *t* | *p***-value** |
| --- | --- | --- | --- | --- |
| Self-rated percentile [vs. 50] | 62.23 (0.93) | [60.40, 64.06] | 13.11 | *<* .001 |
| Confidence (number correct) - Actual –0.48 (0.18) [–0.83, –0.12] | | | –2.65 | .008 |

Table S8: Confidence by performance

Self-rated percentile Confidence (number correct)

Discernment score 6.2830*** (1.9504)

Actual number correct 0.2853***

(0.0755)

| Constant | 59.6641*** (5.1272) | 7.6539*** (1.3257) |
| --- | --- | --- |
| Controls | yes | yes |
| *R*2 | 0.12 | 0.11 |
| N | 463 | 590 |

OLS models with survey weights. * *p <* .05, ** *p <* .01, *** *p <* .005 (two-sided). Models include a standard set of covariates: age, female, college education, nonwhite racial background, and cancer history.

Table S9: Unweighted summary statistics for self-rated percentile and relative overconfidence by actual discernment quartile

Quartile *N* Self-Rated Percentile Overconfidence (Relative)

*M SD* Min Max *M SD* Min Max

1 114 59.82 21.70 1 100 46.82 23.40 -13 98

2 96 57.24 18.22 1 91 19.33 19.67 -35 57

3 126 59.50 19.56 3 100 -2.24 21.03 -52 49

4 127 70.87 17.81 11 100 -17.32 16.48 -71 17

Table S10: Unweighted summary statistics for confidence (number correct) and absolute overcon- fidence by actual number correct quartile

Quartile (Range, *M*) *N* Confidence (Number Correct) Overconfidence (Absolute)

*M SD* Min Max *M SD* Min Max

| 1 (3-8, *M* = 6.77) | 158 | 8.78 | 4.49 | 0 | 18 | 2.01 | 4.63 | -8 | 13 |
| --- | --- | --- | --- | --- | --- | --- | --- | --- | --- |
| 2 (9-10, *M* = 9.55) | 141 | 9.80 | 3.95 | 2 | 18 | 0.26 | 4.03 | -8 | 9 |
| 3 (11-13, *M* = 11.82) | 182 | 9.83 | 3.81 | 0 | 18 | -1.99 | 3.82 | -13 | 7 |
| 4 (14-17, *M* = 14.94) | 109 | 12.45 | 3.24 | 3 | 18 | -2.50 | 3.19 | -13 | 4 |

## Behavioral correlates

Table S11: Exposure to all health sites across overconfidence (relative) and perceived and actual percentile discernment

|  | Exposure (binary) | Exposure (count) | | |
| --- | --- | --- | --- | --- |
| Overconfidence (relative) | 0.0004 | -0.1874 | | |
|  | (0.0007) | (0.1022) | | |
| Actual percentile  Constant | 0.7874*** | -0.0000  (0.0008)  0.7641*** | 12.3362* | 0.2627* (0.1089)  -2.2592 |
|  | (0.0843) | (0.0995) | (5.7431) | (9.8542) |
| Controls | yes | yes | yes | yes |
| *R*2 | 0.04 | 0.05 | 0.07 | 0.07 |
| N | 407 | 528 | 407 | 528 |

OLS models with survey weights. * *p <* .05, ** *p <* .01, *** *p <* .005 (two-sided). Models include a standard set of covariates: age, female, college education, nonwhite racial background, and cancer history.

Table S12: Exposure to low-credibility health sites across perceived and actual percentile discern- ment (separate models)

|  | Exposure (binary) | Exposure (count) | | |
| --- | --- | --- | --- | --- |
| Self-rated percentile | -0.0013 | -0.0193 | | |
|  | (0.0012) | (0.0150) | | |
| Actual percentile  Constant | 0.0278 | -0.0025*** (0.0007)  0.0903* | 1.1601 | -0.0173*** (0.0054)  0.8793* |
|  | (0.0878) | (0.0436) | (1.0268) | (0.3666) |
| Controls | yes | yes | yes | yes |
| *R*2 | 0.10 | 0.12 | 0.05 | 0.06 |
| N | 407 | 528 | 407 | 528 |

OLS models with survey weights. * *p <* .05, ** *p <* .01, *** *p <* .005 (two-sided). Models include a standard set of covariates: age, female, college education, nonwhite racial background, and cancer history.

A15

Table S13: Exposure to low-credibility health sites and belief in cancer risk factors across residu- alized perceived ability

|  | Exposure (binary) | Exposure (count) | False items | True items | Diff. score |
| --- | --- | --- | --- | --- | --- |
| Residualized perceived ability | -0.0004 | -0.0135 | 0.0033 | 0.0024 | -0.0009 |
|  | (0.0013) | (0.0144) | (0.0024) | (0.0020) | (0.0029) |
| Constant | -0.0561 | -0.0679 | 2.1826*** | 3.1191*** | 1.0665*** |
|  | (0.0402) | (0.1626) | (0.1442) | (0.1372) | (0.1695) |
| Controls | yes | yes | yes | yes | yes |
| Item fixed effects |  |  | yes | yes |  |
| *R*2 | 0.10 | 0.04 | 0.09 | 0.08 | 0.08 |
| N | 407 | 407 | 3241 | 3241 | 463 |

OLS models with survey weights. * *p <* .05, ** *p <* .01, *** *p <* .005 (two-sided). Models include a standard set of covariates: age, female, college education, nonwhite racial background, and cancer history.

Table S14: Belief in cancer risk factors across perceived and actual percentile discernment (separate models)

|  | False items | True items | | Diff. score | | |
| --- | --- | --- | --- | --- | --- | --- |
| Self-rated percentile | 0.0003 | 0.0042* | | 0.0039 | | |
|  | (0.0024) | (0.0020) | | (0.0029) | | |
| Actual percentile | -0.0083*** | | 0.0050*** | | 0.0133*** | |
|  | (0.0013) | | (0.0013) | | (0.0015) | |
| Constant | 2.1694*** | 2.7265*** | 2.8513*** | 2.7918*** | 0.8118*** | 0.1913 |
|  | (0.2191) | (0.1594) | (0.1673) | (0.1464) | (0.2532) | (0.1866) |
| Controls | yes | yes | yes | yes | yes | yes |
| Item fixed effects | yes | yes | yes | yes |  |  |
| R2 | 0.09 | 0.13 | 0.09 | 0.10 | 0.09 | 0.28 |
| N | 3241 | 4151 | 3241 | 4151 | 463 | 593 |

OLS models with survey weights. * *p <* .05, ** *p <* .01, *** *p <* .005 (two-sided). Models include a standard set of covariates: age, female, college education, nonwhite racial background, and cancer history.

## Comparison with Lyons et al. 2021

Table S15: Exposure to low-credibility health sites and belief in cancer risk factors (standardized coefficients): Current study

|  | Exposure (binary) | Misperception (diff.) | | | | |
| --- | --- | --- | --- | --- | --- | --- |
| Overconfidence | 0.05* | -0.38*** | | | | |
|  | (0.03) | (0.07) | | | | |
| Self-rating | -0.01 | |  | -0.01 | |  |
|  | (0.03) | |  | (0.07) | |  |
| Actual percentile | -0.07*** | |  | 0.48*** | |  |
|  | (0.02) | |  | (0.06) | |  |
| Residualized self-rating |  | | -0.01 |  | | -0.02 |
|  |  | | (0.03) |  | | (0.07) |
| Constant | -0.05 | -0.03 | -0.06 | 0.46* | 0.36 | 0.51* |
|  | (0.04) | (0.04) | (0.04) | (0.20) | (0.21) | (0.21) |
| Controls | yes | yes | yes | yes | yes | yes |
| *R*2 | 0.12 | 0.14 | 0.10 | 0.20 | 0.27 | 0.08 |
| N | 407 | 407 | 407 | 463 | 463 | 463 |

OLS models with survey weights. * *p <* .05, ** *p <* .01, *** *p <* .005 (two-sided). Models include a standard set of covariates: age, female, college education, nonwhite racial background, and cancer history. For misperception difference score outcomes, entries are standardized betas; outcome and predictor variables are standardized prior to modeling. For exposure outcomes, we do not standardize the binary measure because of issues associated with doing so. However, we standardize all predictors; coefficients represent change in the outcome for a one-standard-deviation change in the predictor.

Table S16: Exposure to dubious news sites and belief in political misperceptions (standardized coefficients): Lyons et al. (2021) dataset

|  | Exposure (binary) | Misperception (diff.) | | | | |
| --- | --- | --- | --- | --- | --- | --- |
| Overconfidence | 0.02** | -0.13*** | | | | |
|  | (0.01) | (0.02) | | | | |
| Self-rating | 0.01 | |  | -0.02 | |  |
|  | (0.01) | |  | (0.02) | |  |
| Actual percentile | -0.01* | |  | 0.16*** | |  |
|  | (0.01) | |  | (0.02) | |  |
| Residualized self-rating |  | | 0.01 |  | | -0.04 |
|  |  | | (0.01) |  | | (0.02) |
| Constant | -0.11*** | -0.11*** | -0.09** | -1.02*** | -0.88*** | -1.13*** |
|  | (0.03) | (0.03) | (0.03) | (0.11) | (0.12) | (0.11) |
| Controls | yes | yes | yes | yes | yes | yes |
| *R*2 | 0.08 | 0.08 | 0.08 | 0.15 | 0.16 | 0.14 |
| N | 2701 | 2701 | 2701 | 2904 | 2904 | 2904 |

OLS models with survey weights. * *p <* .05, ** *p <* .01, *** *p <* .005 (two-sided). Models include a standard set of covariates: age, female, college education, nonwhite racial background, Democrat, Republican, political interest, and political knowledge. For misperception difference score outcomes, entries are standardized betas; outcome and predictor variables are standardized prior to modeling. For exposure outcomes, we do not standardize the binary measure because of issues associated with doing so. However, we standardize all predictors; coefficients represent change in the outcome for a one-standard-deviation change in the predictor.

Table S17: OLS models for common outcomes pooling Lyons et al. (2021) and current study (main effects) [unweighted]

|  | Exposure (binary) | Misp. (diff.) | | | | |
| --- | --- | --- | --- | --- | --- | --- |
| Overconfidence | 0.02*** | -0.22*** | | | | |
|  | (0.00) | (0.02) | | | | |
| Self-rating |  | 0.02*** |  |  | 0.04** |  |
|  |  | (0.00) |  |  | (0.02) |  |
| Actual percentile |  | -0.02*** |  |  | 0.30*** |  |
|  |  | (0.01) |  |  | (0.02) |  |
| Residualized self-rating |  |  | 0.02*** |  |  | 0.03 |
|  |  |  | (0.00) |  |  | (0.02) |
| Study (1 = current) | 0.09*** | 0.09*** | 0.09*** | 0.06 | 0.04 | 0.06 |
|  | (0.01) | (0.01) | (0.01) | (0.05) | (0.05) | (0.05) |
| Constant | -0.01 | -0.01 | -0.00 | -0.18** | -0.13* | -0.19*** |
|  | (0.02) | (0.02) | (0.02) | (0.06) | (0.06) | (0.07) |
| Controls | yes | yes | yes | yes | yes | yes |
| *R*2 | 0.03 | 0.03 | 0.03 | 0.08 | 0.13 | 0.04 |
| N | 3110 | 3110 | 3110 | 3370 | 3370 | 3370 |

OLS models. * *p <* .05, ** *p <* .01, *** *p <* .005 (two-sided). Models include a standard set of covariates common across the two studies: age, female, college education, nonwhite racial background. For misperception difference score outcomes, entries are standardized betas; outcome and predictor variables are standardized prior to modeling. For exposure outcomes, we do not standardize the binary measure because of issues associated with doing so. However, we standardize all predictors; coefficients represent change in the outcome for a one-standard-deviation change in the predictor.

Table S18: OLS models for common outcomes pooling Lyons et al. (2021) and current study (main effects) [weighted]

|  | Exposure (binary) | Misp. (diff.) | | | | |
| --- | --- | --- | --- | --- | --- | --- |
| Overconfidence | -0.00 | -0.27* | | | | |
|  | (0.02) | (0.12) | | | | |
| Self-rating |  | 0.01 |  |  | -0.02 |  |
|  |  | (0.01) |  |  | (0.09) |  |
| Actual percentile |  | 0.01 |  |  | 0.33*** |  |
|  |  | (0.03) |  |  | (0.11) |  |
| Residualized self-rating |  |  | 0.01 |  |  | -0.01 |
|  |  |  | (0.01) |  |  | (0.10) |
| Study (1 = current) | 0.03 | 0.03 | 0.03 | 0.12 | 0.05 | 0.09 |
|  | (0.04) | (0.04) | (0.04) | (0.27) | (0.26) | (0.26) |
| Constant | -0.02 | -0.02 | -0.02 | 0.03 | 0.13 | 0.01 |
|  | (0.06) | (0.06) | (0.06) | (0.30) | (0.28) | (0.28) |
| Controls | yes | yes | yes | yes | yes | yes |
| *R*2 | 0.02 | 0.02 | 0.02 | 0.07 | 0.09 | 0.01 |
| N | 3110 | 3110 | 3110 | 3370 | 3370 | 3370 |

OLS models with survey weights. * *p <* .05, ** *p <* .01, *** *p <* .005 (two-sided). Models include a standard set of covariates common across the two studies: age, female, college education, nonwhite racial background. For misperception difference score outcomes, entries are standardized betas; outcome and predictor variables are standardized prior to modeling. For exposure outcomes, we do not standardize the binary measure because of issues associated with doing so. However, we standardize all predictors; coefficients represent change in the outcome for a one-standard-deviation change in the predictor.

Table S19: Effect comparison between current study and Lyons et al. (2021) dataset (pooled) [unweighted]

|  | Exposure (binary) |  |  | Misp. (diff.) |  |  |
| --- | --- | --- | --- | --- | --- | --- |
| Study (1 = current) | 0.09*** | 0.10*** | 0.09*** | 0.06 | 0.02 | 0.06 |
|  | (0.01) | (0.01) | (0.01) | (0.05) | (0.05) | (0.05) |
| Overconfidence | 0.02*** |  |  | -0.18*** |  |  |
|  | (0.01) |  |  | (0.02) |  |  |
| Overconf. *×* study | 0.03 |  |  | -0.24*** |  |  |
|  | (0.01) |  |  | (0.05) |  |  |
| Self-rating |  | 0.02*** |  |  | 0.06*** |  |
|  |  | (0.01) |  |  | (0.02) |  |
| Actual percentile |  | -0.01* |  |  | 0.26*** |  |
|  |  | (0.01) |  |  | (0.02) |  |
| Actual *×* study |  | -0.04*** |  |  | 0.25*** |  |
|  |  | (0.01) |  |  | (0.05) |  |
| Self-rating *×* study |  | -0.03 |  |  | -0.11* |  |
|  |  | (0.01) |  |  | (0.05) |  |
| Residualized self-rating |  |  | 0.02*** |  |  | 0.04* |
|  |  |  | (0.01) |  |  | (0.02) |
| Residualized *×* study |  |  | -0.02 |  |  | -0.12* |
|  |  |  | (0.01) |  |  | (0.05) |
| Constant | -0.01 | -0.00 | -0.00 | -0.18*** | -0.13* | -0.19*** |
|  | (0.02) | (0.02) | (0.02) | (0.06) | (0.06) | (0.07) |
| Controls | yes | yes | yes | yes | yes | yes |
| *R*2 | 0.03 | 0.03 | 0.03 | 0.09 | 0.13 | 0.04 |
| N | 3110 | 3110 | 3110 | 3370 | 3370 | 3370 |

OLS models. * *p <* .05, ** *p <* .01, *** *p <* .005 (two-sided). Models include a standard set of covariates common across the two studies: age, female, college education, nonwhite racial background. For misperception difference score outcomes, entries are standardized betas; outcome and predictor variables are standardized prior to modeling. For exposure outcomes, we do not standardize the binary measure because of issues associated with doing so. However, we standardize all predictors; coefficients represent change in the outcome for a one-standard-deviation change in the predictor. The interaction term reflects the difference in the effect between the studies.

Table S20: Effect comparison between current study and Lyons et al. (2021) dataset (pooled) [weighted]

|  | Exposure (binary) |  |  | Misp. (diff.) |  |  |
| --- | --- | --- | --- | --- | --- | --- |
| Study (1 = current) | 0.03 | 0.05 | 0.03 | 0.16 | 0.08 | 0.09 |
|  | (0.04) | (0.04) | (0.04) | (0.25) | (0.23) | (0.26) |
| Overconfidence | -0.02 |  |  | -0.12 |  |  |
|  | (0.03) |  |  | (0.08) |  |  |
| Overconf. *×* study | 0.06 |  |  | -0.44 |  |  |
|  | (0.04) |  |  | (0.28) |  |  |
| Self-rating |  | 0.02 |  |  | -0.02 |  |
|  |  | (0.01) |  |  | (0.07) |  |
| Actual percentile |  | 0.04 |  |  | 0.16 |  |
|  |  | (0.04) |  |  | (0.09) |  |
| Actual *×* study |  | -0.08 |  |  | 0.53* |  |
|  |  | (0.05) |  |  | (0.26) |  |
| Self-rating *×* study |  | -0.00 |  |  | -0.05 |  |
|  |  | (0.03) |  |  | (0.25) |  |
| Residualized self-rating |  |  | 0.02 |  |  | -0.01 |
|  |  |  | (0.01) |  |  | (0.07) |
| Residualized *×* study |  |  | -0.01 |  |  | 0.01 |
|  |  |  | (0.03) |  |  | (0.31) |
| Constant | -0.01 | -0.03 | -0.02 | -0.04 | 0.02 | 0.01 |
|  | (0.06) | (0.06) | (0.06) | (0.27) | (0.26) | (0.28) |
| Controls | yes | yes | yes | yes | yes | yes |
| *R*2 | 0.03 | 0.04 | 0.02 | 0.10 | 0.14 | 0.01 |
| N | 3110 | 3110 | 3110 | 3370 | 3370 | 3370 |

OLS models with survey weights. * *p <* .05, ** *p <* .01, *** *p <* .005 (two-sided). Models include a standard set of covariates common across the two studies: age, female, college education, nonwhite racial background. For misperception difference score outcomes, entries are standardized betas; outcome and predictor variables are standardized prior to modeling. For exposure outcomes, we do not standardize the binary measure because of issues associated with doing so. However, we standardize all predictors; coefficients represent change in the outcome for a one-standard-deviation change in the predictor. The interaction term reflects the difference in the effect between the studies.

# Other preregistered results

## Who is overconfident?

Next we provide analyses of attitudinal and dispositional correlates of overconfidence. We used a full set of potential correlates in an “omnibus” OLS regression model: health literacy, digital literacy, anti-expert views, conspiracism, and feelings toward a range of relevant entities (pharma- ceutical companies, doctors, science, the media, and social media). We also include the standard set of controls as in all previous models: age, female, college education, nonwhite racial background. Note that we use dichotomous indicators for age groups because a linear term may mask nonlinear- ities and studies examining age differences in news consumption stress that the oldest sub-group of users (typically those 60+) are most likely to consume low credibility news or media content (Guess et al., [2020](#_bookmark84); Moore et al., [2023](#_bookmark88)). We use a binary indicator for college education to capture a key distinction in educational attainment following the target study and other work in the field (Guess et al., [2020](#_bookmark84); Lyons et al., [2021](#_bookmark87)).

We found that three measures were associated with the measure of relative overconfidence (Ta- ble [S21](#_bookmark64)). Specifically, respondents with stronger anti-expert views (*b* = 9.83, *SE* = 2.36, *p <* .001), greater conspiracism (*b* = 5.49, *SE* = 1.96, *p* = .005), and more negative feelings toward science (*b* = *−*0.25, *SE* = .09, *p* = .009) were more overconfident in their performance relative to others. Or, viewed differently, those with more favorable views toward experts and science, and less con- spiracism, exhibited less overconfidence in their ability to discern accurate and inaccurate cancer information. Notably, when examining correlates of self-rated and actual discernment performance separately in Table [S21](#_bookmark64), we found that anti-expert views were associated with both higher self-rating (*b* = 3.97, *SE* = 1.85, *p* = .033) and worse actual performance (*b* = *−*5.48, *SE* = 1.96, *p* = .005), while conspiracism and feelings toward science were only associated with actual performance (con- spiracism *b* = *−*4.64, *SE* = 1.61, *p* = .004; feelings toward science *b* = .28, *SE* = .07, *p <* .001, re- spectively). We show descriptive means of relative overconfidence across these correlated measures in Figure [S2](#_bookmark63).

Based on these findings, we explored potential differences between the profiles of relative and absolute measures of overconfidence before moving onto examining their associations with be- havioral correlates. Measures of overconfidence may be driven disproportionately by differences in one’s ability to recognize one veracity category rather than another (i.e., accurate or inaccurate items). In Figure [S3](#_bookmark66) and Table [S22](#_bookmark65), we disaggregated these components to show the relative contri- bution of each veracity category. The percentile-based measure of overconfidence was much more strongly shaped by ratings of inaccurate items (that is, mistakenly rating such headlines as accurate) than by ratings of accurate items. Meanwhile, the contribution of each type to the absolute measure was more balanced. Therefore, the relative measure tended to better capture the thinking styles and tendencies of those who *affirmatively* endorse misinformation.

A22

50

40

Overconfidence

30

20

10

0

-10

-20

50

40

Overconfidence

30

20

10

0

-10

-20


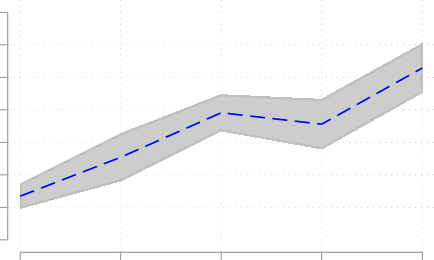


1 2 3 4 5

5 quintiles of anti-expert views


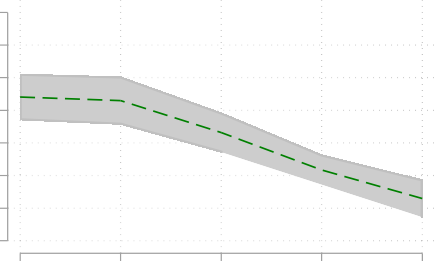


1 2 3 4 5

5 quintiles of warmth toward science

50

40

Overconfidence

30

20

10

0

-10

-20


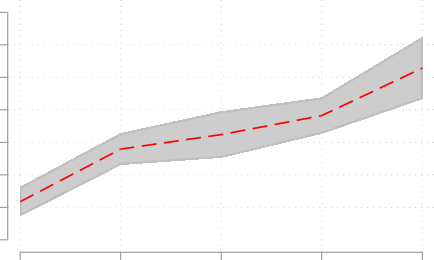


1 2 3 4 5

5 quintiles of conspiracism

Figure S2: **Overconfidence (relative) across anti-expert views, conspiracism, and warmth to- ward science.** Figure shows descriptive means with 95% confidence intervals. Overconfidence (relative) is computed as (self-rated percentile) – (actual percentile in discernment), with discern- ment being a measure of performance in distinguishing accurate from inaccurate cancer informa- tion. Anti-expert views, conspiracism, and warmth toward science are divided into quintiles for visualization. For full modeled results include a set of covariates, see Table [S21](#_bookmark64).

Table S21: Correlates of Overconfidence and Components

Overconfidence measure

|  | Relative | Absolute |  | Self-rating | Actual Percentile |  |
| --- | --- | --- | --- | --- | --- | --- |
| Age 30–44 | 3.93 | -0.33 |  | -4.46 | -8.78* |  |
|  | (4.91) | (0.86) |  | (2.85) | (4.44) |  |
| Age 45–59 | 5.10 | -1.06 |  | -5.74 | -11.03* |  |
|  | (5.25) | (0.91) |  | (3.03) | (4.42) |  |
| Age 60+ | 5.12 | 0.22 |  | -6.51* | -11.49** |  |
|  | (5.16) | (0.84) |  | (2.88) | (4.38) |  |
| Female | -3.99 | -1.23*** |  | -6.35*** | -2.08 |  |
|  | (3.06) | (0.44) |  | (2.09) | (2.46) |  |
| College | -2.88 | -0.18 |  | 4.17 | 7.60*** |  |
|  | (3.18) | (0.47) |  | (2.36) | (2.38) |  |
| Nonwhite | 5.78 | 0.18 |  | -0.39 | -4.83 |  |
|  | (3.80) | (0.52) |  | (2.53) | (2.87) |  |
| Cancer history | 1.35 | 0.06 |  | 8.32* | 6.68 |  |
|  | (5.68) | (0.57) |  | (3.22) | (4.32) |  |
| Health literacy | -3.67 | 0.48 |  | 3.55* | 4.67* |  |
|  | (2.39) | (0.35) |  | (1.43) | (2.05) |  |
| Low-end digital literacy | -1.87 | -0.30 |  | -2.23 | -0.34 |  |
|  | (2.39) | (0.34) |  | (1.51) | (1.85) |  |
| Anti-expert views | 9.83*** | 0.47 |  | 3.97* | -5.48** |  |
|  | (2.36) | (0.35) |  | (1.85) | (1.96) |  |
| Conspiracism | 5.49** | 0.30 |  | 0.90 | -4.64*** |  |
|  | (1.96) | (0.31) |  | (1.35) | (1.61) |  |
| Affect to pharma | 0.06 | -0.00 |  | -0.00 | -0.08 |  |
|  | (0.07) | (0.01) |  | (0.05) | (0.05) |  |
| Affect to doctors | -0.11 | -0.02 |  | 0.08 | 0.18** |  |
|  | (0.10) | (0.01) |  | (0.06) | (0.07) |  |
| Affect to science | -0.25** | -0.01 |  | 0.00 | 0.28*** |  |
|  | (0.09) | (0.01) |  | (0.06) | (0.07) |  |
| Affect to media | 0.08 | -0.01 |  | 0.14* | 0.09 |  |
|  | (0.08) | (0.01) |  | (0.05) | (0.05) |  |
| Affect to social media | 0.03 | -0.00 |  | -0.01 | -0.09 |  |
|  | (0.07) | (0.01) |  | (0.06) | (0.06) |  |
| Constant | 5.91 | -0.76 |  | 34.63*** | 38.22*** |  |
|  | (15.35) | (2.19) |  | (10.45) | (12.92) |  |
| *R*2 | 0.36 | 0.12 |  | 0.15 | 0.43 |  |
| N | 442 | 537 |  | 442 | 538 |  |

OLS models with survey weights. * *p <* .05, ** *p <* .01, *** *p <* .005 (two-sided). 18–29 is the reference category for age.

Table S22: Correlations of overconfidence with accuracy ratings

| Inaccurate headline rating (avg.) | 0.6503 | 0.1072 |
| --- | --- | --- |
| Accurate headline rating (avg.) | -0.2761 | -0.2440 |

### Overconf. (relative) Overconf. (absolute)

4 4


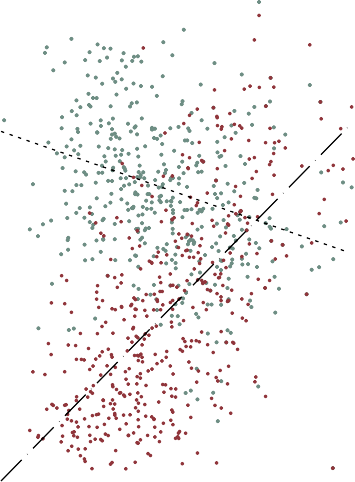

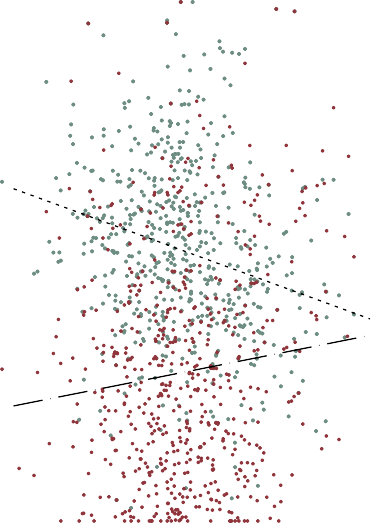


3 3

Accuracy rating

Accuracy rating

2

2

1

-100 -50 0 50 100

Overconfidence (relative)

1

-15 -10 -5 0 5 10 15

Overconfidence (absolute)

Accurate cancer news Inaccurate cancer news

Figure S3: **Overconfidence across performance on accurate and inaccurate cancer news head- lines**

## Effects of interventions on overconfidence

The study used a 3 (intervention treatment: a generic media literacy intervention, a health-focused media literacy intervention, control) × 2 (outcome measure: perceived accuracy, sharing intent) design. Our selected generic media literacy intervention (News Tips) has been tested in several other studies and replicates Facebook’s “Tips to Spot False News,” which were developed in col- laboration with the nonprofit First Draft and subsequently promoted at the top of users’ News Feeds in 14 countries in April 2017 and printed in full-page newspaper ads in the USA, UK, France, Ger- many, Mexico, and India. Our health-focused media literacy intervention (BOAST), meanwhile, is based on a set of tips developed by the nonprofit organization Facing Our Risk of Cancer Empow- ered (FORCE) (Lyons et al., [2024](#_bookmark86)). We preregistered a set of analyses to examine whether these treatments affect (over)confidence. We find limited evidence that these treatments effects these outcomes.

.

Table S23: Effects of media literacy interventions on (over)confidence

|  | Self-rated percentile | Confidence (number correct) | Overconfidence (relative) | Overconfidence (absolute) |
| --- | --- | --- | --- | --- |
| BOAST | 1.7237 | 0.5033 | 1.9333 | 1.0859* |
|  | (2.2200) | (0.4052) | (3.5396) | (0.4232) |
| News tips | 3.9085 | 0.5077 | 0.1456 | 0.1415 |
| Constant | (2.4588) 60.3782*** | (0.4359)  9.6667*** | (3.7609)  9.4744*** | (0.4641)  -0.8889** |
|  | (1.7701) | (0.3033) | (2.6453) | (0.3229) |
| *R*2 | 0.01 | 0.00 | 0.00 | 0.01 |
| N | 463 | 593 | 463 | 590 |

OLS models. * *p <* .05, ** *p <* .01, *** *p <* .005 (two-sided).

# Robustness tests

## Replication using control condition data

Because the media literacy interventions described in the previous section may have influenced other measurements as well, this section 1) tests whether there were any treatment effects on post- treatment measures and 2) tests whether the primary descriptive results for (over)confidence repli- cate using data from the control condition. Table [S24](#_bookmark71) shows no treatment effects on discernment, number correct, or post-treatment visits to low-credibility sites (note that cancer misperceptions were measured pre-treatment).

Table [S25](#_bookmark72) shows high correspondence in means for all key outcome measures relating to dis- cernment and confidence, and Tables [S26](#_bookmark74) and [S27](#_bookmark75) show this extends to confidence and overcon- fidence across quartiles of performance. Figure [S4](#_bookmark73) therefore shows similar correlation of perfor- mance and confidence items, with Figure [S5](#_bookmark76) replicating a “Dunning-Kruger” pattern correspond- ing to the one depicted in the main text. Lastly, the key correlates of overconfidence — anti-expert views, conspiracism, and warmth toward science – show similar degrees of correlation in control condition data, especially when looking at bivariate relations (Table [S29](#_bookmark78) and Figure [S6](#_bookmark79)).

Table S24: Effects of media literacy interventions on discernment and exposure to low-credibility health sites (full sample)

|  | Discernment | Number correct | Exposure (bin) | Exposure (count) |
| --- | --- | --- | --- | --- |
| BOAST | -0.0043 | -0.5556 | 0.0188 | 0.0075 |
|  | (0.0695) | (0.2906) | (0.0202) | (0.1401) |
| News tips | 0.1296 | 0.4084 | 0.0136 | -0.0298 |
| Constant | (0.0750)  0.6536*** | (0.3043)  10.5556*** | (0.0197)  0.0676*** | (0.1286)  0.3239*** |
|  | (0.0508) | (0.2088) | (0.0133) | (0.0953) |
| *R*2 | 0.01 | 0.02 | 0.00 | 0.00 |
| N | 593 | 590 | 1059 | 1059 |

OLS models. * *p <* .05, ** *p <* .01, *** *p <* .005 (two-sided).

Table S25: Unweighted summary statistics for outcomes (control condition)

| **Variable** | ***M*** | ***SD*** | **Min** | **Max** |
| --- | --- | --- | --- | --- |
| Self-rated percentile | 60.38 | 22.11 | 1 | 100 |
| Confidence (number correct) | 10.34 | 4.18 | 0 | 18 |
| Actual number correct | 10.73 | 3.04 | 3 | 16 |
| Overconfidence (relative) | 11.09 | 32.44 | -67 | 98 |
| Overconfidence (absolute) | -0.39 | 4.56 | -13 | 11 |

100

75

Self-Rated Percentile

50

25

0


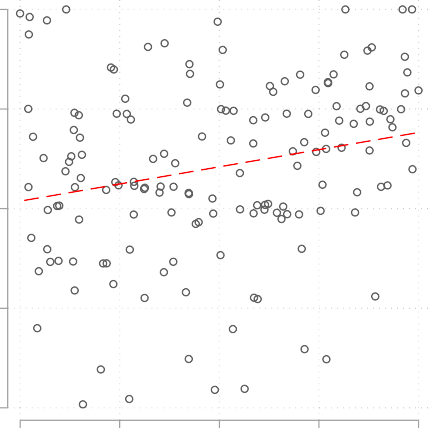


0 25 50 75 100

Actual Discernment Percentile

18


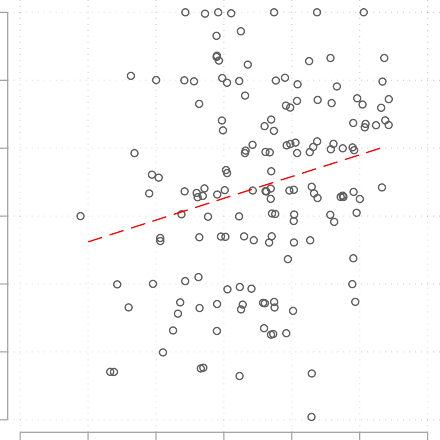
15

Confidence (Number Correct)

12

9

6

3

0

0 3 6 9 12 15 18

Actual Number Correct

Figure S4: **Scatterplots of confidence and actual performance measures (control condition).** Actual discernment is difference score of performance in distinguishing accurate from inaccurate cancer information, while actual number correct is a sum of correct judgments.

Table S26: Unweighted summary statistics for self-rated percentile and relative overconfidence by actual discernment quartile (control condition)

Quartile *N* Self-Rated Percentile Overconfidence (Relative)

*M SD* Min Max *M SD* Min Max

1 42 57.10 23.74 1 100 44.76 25.87 -13 98

2 36 54.33 21.80 1 91 17.00 23.27 -46 57

3 39 57.72 21.00 5 96 -4.41 22.63 -58 45

4 39 72.15 17.64 11 100 -15.13 17.93 -67 19

Table S27: Unweighted summary statistics for confidence (number correct) and absolute overcon- fidence by actual number correct quartile (control condition)

Quartile *N* Confidence (Number Correct) Overconfidence (Absolute)

*M SD* Min Max *M SD* Min Max

| 1 (3-9, *M* = 7.44) | 55 | 9.89 4.73 | 2 | 18 | 2.45 4.45 | -6 | 11 |
| --- | --- | --- | --- | --- | --- | --- | --- |
| 2 (10-11, *M* = 10.59) | 39 | 9.49 4.06 | 2 | 18 | -1.10 4.12 | -8 | 7 |
| 3 (12-13, *M* = 12.41) | 29 | 10.55 4.13 | 0 | 18 | -1.86 4.18 | -13 | 5 |
| 4 (14-16, *M* = 14.91) | 33 | 11.91 2.92 | 5 | 18 | -3.00 2.86 | -10 | 3 |

100

75

Self-rated percentile

50

25

0


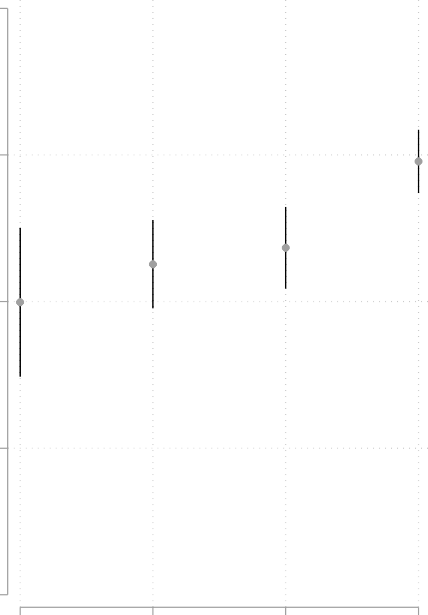


1 2 3 4

Actual discernment quartile

18


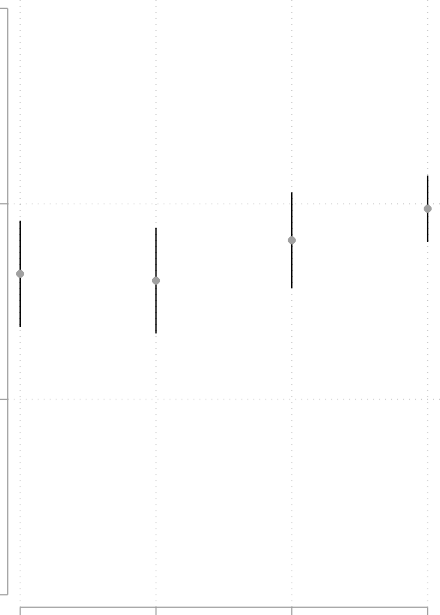
12

Confidence (number correct)

6

0

1 2 3 4

Actual number correct quartile

Figure S5: **Confidence (relative and absolute) across quartiles of actual performance (control condition).** Actual discernment is difference score of performance in distinguishing accurate from inaccurate cancer information, while actual number correct is a sum of correct judgments.

Table S28: Correlates of overconfidence (control condition)

Overconfidence (relative) Overconfidence (absolute)

Age 30-44 7.7093 -1.6109

(7.0871) (1.3992)

Age 45-59 5.5038 -3.6087**

(6.6451) (1.3808)

Age 60+ 5.9638 -1.9718

(7.3707) (1.3397)

Female -6.3061 -0.9941

(4.4211) (0.7828)

College 0.5316 -1.0028

(5.2073) (0.8655)

Nonwhite 8.6672 -0.6022

(4.8809) (0.8676)

Cancer history 8.1320 0.2038

(5.9184) (1.1471)

Health literacy -0.2246 1.7499*

(3.6284) (0.7243)

Low-end digital literacy -1.1916 -0.1714

(4.3563) (0.7034)

Anti-expert views 1.5494 0.6066

(3.1592) (0.5882)

Conspiracism 3.6734 -0.2108

(2.7084) (0.4889)

Affect to pharma 0.1570 0.0357

(0.1000) (0.0195)

affect to doctors -0.3792* -0.0216

(0.1532) (0.0232)

Affect to science -0.4981*** -0.0638***

(0.1657) (0.0207)

Affect to media 0.0979 0.0014

(0.1008) (0.0164)

Affect to social media 0.0897 0.0019

(0.1072) (0.0165)

| Constant | 40.9632 | -1.3373 |
| --- | --- | --- |
|  | (24.6294) | (4.3631) |
| *R*2 | 0.42 | 0.24 |
| N | 146 | 146 |

OLS models with survey weights. * *p <* .05, ** *p <* .01, *** *p <* .005 (two-sided). 18-29 is the reference category for age.

Table S29: Bivariate correlations of overconfidence (relative) and key individual difference mea- sures

Full Sample Control Condition

| **Variable** | *r* | *p* |  | *r* | *p* |  |
| --- | --- | --- | --- | --- | --- | --- |
| Anti-expert views | 0.48 | *<* 0.001 |  | 0.47 | *<* 0.001 |  |
| Conspiracism  Warmth toward science | 0.44  *−*0.38 | *<* 0.001  *<* 0.001 |  | 0.42  *−*0.40 | *<* 0.001  *<* 0.001 |  |

50

40

Overconfidence

30

20

10

0

-10

-20

50

40

Overconfidence

30

20

10

0

-10

-20


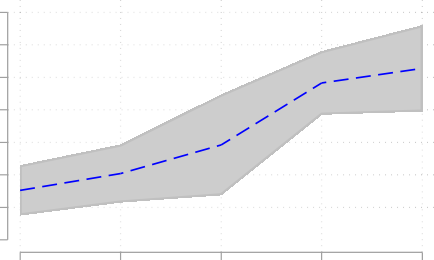


1 2 3 4 5

5 quintiles of anti-expert views


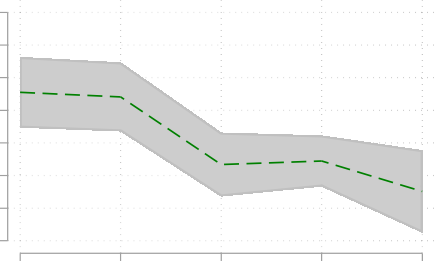


1 2 3 4 5

5 quintiles of warmth toward science

50

40

Overconfidence

30

20

10

0

-10

-20


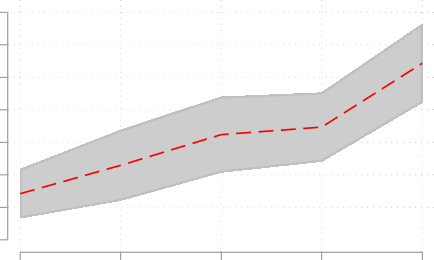


1 2 3 4 5

5 quintiles of conspiracism

Figure S6: **Overconfidence (relative) across anti-expert views, conspiracism, and warmth to- ward science (control condition).** Figure shows descriptive means with 95% confidence intervals. Overconfidence (relative) is computed as (self-rated percentile) – (actual percentile in discernment), with discernment being a measure of performance in distinguishing accurate from inaccurate cancer information. Anti-expert views, conspiracism, and warmth toward science are divided into quin- tiles for visualization. For full modeled results include a set of covariates, see Table [S28](#_bookmark77).

## Alternative treatment of missing data

As indicated in the Methods section, relative confidence (self-rated percentile) was measured on a 1-100 scale, on which respondents were also able to select “don’t know” as a response option, which we treat as missing (*n* = 130 DKs) in our primary analyses. Here, we use an alternative version of this variable that treats DKs as 50 (midpoint) on the scale. Below, we report the results for our key relative overconfidence models (exposure and misperception outcomes) using this calculation. As in our primary results in the main text, we find that this measure of relative overconfidence is associated with exposure to low-credibility health sites and cancer misperception outcomes, but this is primarily accounted for by actual performance.

Table S30: Exposure to low-credibility health sites by (over)confidence (relative) (alternative treat- ment of missing data)

Exposure (binary) Exposure (count)

Overconfidence (relative) recode 0.0019** 0.0094*

(0.0007) (0.0042)

Self-rated percentile recode -0.0002 -0.0105

(0.0012) (0.0122)

Actual percentile -0.0025*** -0.0157***

(0.0007) (0.0047)

| Constant | -0.0626 | 0.0998 | -0.1484 | 1.4307 |
| --- | --- | --- | --- | --- |
|  | (0.0366) | (0.0775) | (0.1446) | (0.9086) |
| Controls | yes | yes | yes | yes |
| *R*2 | 0.11 | 0.12 | 0.04 | 0.07 |
| N | 528 | 528 | 528 | 528 |

OLS models with survey weights. * *p <* .05, ** *p <* .01, *** *p <* .005 (two-sided). 18-29 is the reference category for age. Models include a standard set of covariates: age, female, college education, nonwhite racial background, and cancer history.

Table S31: Cancer misperceptions by overconfidence (relative) (alternative treatment of missing data)

|  | False items | True items | False items | True items | Diff. score | Diff. score |
| --- | --- | --- | --- | --- | --- | --- |
| Overconfidence (relative) recode | 0.0070*** | -0.0027* |  |  | -0.0097*** |  |
|  | (0.0011) | (0.0013) |  |  | (0.0015) |  |
| Self-rated percentile recode | 0.0022 | | | 0.0030 | 0.0008 | |
|  | (0.0022) | | | (0.0020) | (0.0027) | |
| Actual percentile | -0.0086*** | | | 0.0046*** | 0.0132*** | |
|  | (0.0013) | | | (0.0013) | (0.0015) | |
| Constant | 2.2067*** | 3.0919*** | 2.6061*** | 2.6256*** | 1.0111*** | 0.1454 |
|  | (0.1363) | (0.1318) | (0.2160) | (0.1625) | (0.1546) | (0.2478) |
| Controls | yes | yes | yes | yes | yes | yes |
| Item fixed effects | yes | yes | yes | yes |  |  |
| *R*2 | 0.12 | 0.09 | 0.13 | 0.10 | 0.21 | 0.28 |
| N | 4151 | 4151 | 4151 | 4151 | 593 | 593 |

OLS models with survey weights. * *p <* .05, ** *p <* .01, *** *p <* .005 (two-sided). Models include a standard set of covariates: age, female, college education, nonwhite racial background, and cancer history.

# Populated pre-analysis plan

RQ1. To what extent will people who are least accurate at distinguishing between legitimate and false news overrate their ability?

- Tables [S9](#_bookmark47), [S10](#_bookmark48), and [S8](#_bookmark46). T-tests reported in text and Table[S7](#_bookmark45).

RQ2. Is overconfidence positively related to holding misperceptions on cancer?

- Table [2](#_bookmark30)

RQ3: What other variables are associated overconfidence?

- Table [S21](#_bookmark64)

RQ4. What are the effects of interventions on confidence and overconfidence?

- Table [S23](#_bookmark68)

RQ5: Is overconfidence associated with news behavior via trace data?

- Tables [1](#_bookmark28)

# References

Aslett, K., Sanderson, Z., Godel, W., Persily, N., Nagler, J., Bonneau, R., & Tucker, J. A. (2024). Testing the effect of information on discerning the veracity of news in real time. *Journal of* *Experimental Political Science*, *11*(3), 262–276.

Guess, A. M., & Munger, K. (2023). Digital literacy and online political behavior. *Political Science* *Research and Methods*, *11*(1), 110–128. <https://doi.org/10.1017/psrm.2022.17>

Guess, Nyhan, B., & Reifler, J. (2020). Exposure to untrustworthy websites in the 2016 us election.

*Nature human behaviour*, *4*(5), 472–480.

Han, H., Blackburn, A. M., Jeftić, A., Tran, T. P., Stöckli, S., Reifler, J., & Vestergren, S. (2022). Validity testing of the conspiratorial thinking and anti-expert sentiment scales during the covid-19 pandemic across 24 languages from a large-scale global dataset. *Epidemiology &* *Infection*, *150*, e167.

Lyons, King, A. J., & Kaphingst, K. A. (2024). A health media literacy intervention increases skep- ticism of both inaccurate and accurate cancer news among us adults. *Annals of Behavioral* *Medicine*, *58*(12), 820–831.

Lyons, Montgomery, J. M., Guess, A. M., Nyhan, B., & Reifler, J. (2021). Overconfidence in news judgments is associated with false news susceptibility. *Proceedings of the National Academy* *of Sciences*, *118*(23), e2019527118.

Moore, R. C., Dahlke, R., & Hancock, J. T. (2023). Exposure to untrustworthy websites in the 2020 us election. *Nature Human Behaviour*, 1–10.

Pennycook, G., Binnendyk, J., Newton, C., & Rand, D. G. (2021). A practical guide to doing be- havioral research on fake news and misinformation. *Collabra: Psychology*, *7*(1), 25293.

Wallston, K. A., Cawthon, C., McNaughton, C. D., Rothman, R. L., Osborn, C. Y., & Kripalani,

S. (2014). Psychometric properties of the brief health literacy screen in clinical practice.

*Journal of general internal medicine*, *29*, 119–126.
